# KMV-mediated cardiomyocyte-to-endothelial cell signaling drives capillary rarefaction to promote heart failure following pressure overload

**DOI:** 10.7150/thno.104899

**Published:** 2025-03-31

**Authors:** Chenxi Gao, Yudong Xia, Chaofang Li, Tao Zhou, Wenyu Zhang, Hao Cheng, Xiaojin Zhang, Yunhao Yu, Chuanfu Li, Zhengnian Ding, Jun Wu, Li Liu

**Affiliations:** 1 Departments of Geriatrics, First Affiliated Hospital of Nanjing Medical University, Nanjing 210029, China.; 2 Department of Cardiology, First Affiliated Hospital of Nanjing Medical University, Nanjing 210029, China.; 3 Departments of Anesthesiology, First Affiliated Hospital of Nanjing Medical University, Nanjing 210029, China.; 4 Department of Cardiology, Affiliated Nanjing Brain Hospital of Nanjing Medical University, Nanjing 210029, China.; 5 Department of Anesthesiology, First Affiliated Hospital with Wannan Medical College, Wuhu, China.; 6 Departments of Surgery, East Tennessee State University, Johnson City, TN 37614, USA.; 7 Key Laboratory of Targeted Intervention of Cardiovascular Disease, Collaborative Innovation Center for Cardiovascular Disease Translational Medicine, Nanjing Medical University, China.

**Keywords:** heart failure, angiogenesis, cardiomyocyte hypertrophy, endothelial cells, pressure overload, MEOX2, senescence

## Abstract

**Rationale:** Pressure overload (PO)-induced heart failure (HF) is a global health burden with poor outcomes. Cardiomyocyte hypertrophy and capillary rarefaction are two features and drivers of PO-induced HF. Metabolism is altered in hypertrophic cardiomyocytes; however, the metabolites secreted by hypertrophic cardiomyocytes to paracrinally regulate capillary density remain to be identified.

**Methods:** PO-induced HF was established by transverse aortic constriction in mice. Metabolite secretion was examined by nontargeted metabolomics and enzyme-linked immunosorbent assays. Gene expression was examined by RNA-sequencing and immunoblotting. Protein-promoter binding was examined by chromatin immunoprecipitation-PCR. Cardiomyocyte hypertrophy and cardiac capillary density were examined by immunostaining for -actinin and CD31, respectively. *In vitro* angiogenesis was indicated by proliferation, migration, tube formation, and angiogenic factor expression of endothelial cells (ECs). EC senescence was determined by SA--gal staining and p16 and p21 expression.

**Results:** There was a negative correlation between cardiomyocyte size and capillary density in PO-induced failing hearts, and hypertrophic cardiomyocytes paracrinally inhibited angiogenesis of ECs. 3-Methyl-2-oxovaleric acid (KMV) was the most upregulated metabolite secreted by hypertrophic cardiomyocytes. Notably, KMV treatment resulted in EC senescence and angiogenesis inhibition *in vitro* and exaggerated PO-induced EC senescence, capillary rarefaction, and cardiac dysfunction of mice *in vivo*. Additionally, KMV increased expression and nuclear accumulation of mesenchyme homeobox 2 (Meox2) in ECs, whereas Meox2 knockdown diminished KMV-induced EC senescence and angiogenesis inhibition. Furthermore, Meox2 directly bound to the promoter of the senescence-related gene *p21* in ECs, and this binding was enhanced by KMV.

**Conclusions:** The data suggest that hypertrophic cardiomyocytes secrete elevated levels of KMV, which paracrinally increases nuclear accumulation of Meox2 in ECs, where Meox2 binds to the promoter of *p21* and thereby triggers EC senescence and subsequent angiogenesis impairment, ultimately driving capillary rarefaction to promote PO-induced HF. The findings identified KMV as a novel metabolite secreted by hypertrophic cardiomyocytes that triggered EC senescence to drive PO-induced capillary rarefaction and subsequent HF development. Targeting this KMV-mediated cardiomyocyte-to-EC signaling has therapeutic potential in the management of PO-induced HF in patients.

## Introduction

Heart failure (HF) is a major global health burden that affects over 64 million people worldwide and leads to significant morbidity and mortality 1, 2. Although much progress has been made in HF management in recent years, the prognosis remains poor, with a 3-year mortality rate of up to 30-50% 1. Pressure overload (PO), which occurs in conditions such as hypertension and aortic stenosis, is a major cause of HF development 3. PO induces a complex series of adaptive and maladaptive responses in the heart, initially leading to compensatory hypertrophy but eventually progressing to decompensated HF 4, 5. However, the precise mechanisms underlying the transition from compensated hypertrophy to decompensated HF in response to PO remain elusive, which hinders the development of effective therapies.

Patients with PO-induced failing hearts have evidence of capillary rarefaction 6. Capillary rarefaction, which is defined as a reduction of capillary density, is considered a crucial player in the pathogenesis of PO-induced HF. In PO hearts, the imbalance between capillary rarefaction (decreased capillary density) and cardiomyocyte hypertrophy (increased cardiomyocyte size) leads to a mismatch between cardiac oxygen supply and demand, contributing to myocardial hypoxia, fibrosis, and, ultimately, contractile dysfunction 6-8. Although the exact mechanisms underlying capillary rarefaction in failing hearts are not fully understood, recent studies have implicated endothelial cell (EC) senescence as a potential contributor 9, 10. Senescent ECs are characterized by cell cycle arrest and exhibit angiogenesis impairment, barrier dysregulation, and secretory abnormalities. All these changes in senescent ECs result not only in impairment of angiogenesis but also in improper repair of microvascular damage, both of which lead to capillary loss 11, 12. Therefore, targeting EC senescence to maintain an appropriate capillary density is a promising therapeutic intervention to manage PO-induced HF. However, the mechanisms underlying EC senescence and subsequent capillary rarefaction in failing hearts have not been clearly elucidated.

Crosstalk between cardiomyocytes and ECs is essential to maintain cardiac homeostasis and function under normal conditions 13. This crosstalk has also been observed in cardiac pathological conditions. In response to PO, cardiomyocytes undergo hypertrophy, and hypertrophic cardiomyocytes exhibit altered metabolic demands and secrete altered metabolites, which can profoundly and paracrinally influence EC behaviors 14, 15. However, the metabolites that mediate cardiomyocyte-to-EC signaling to modulate EC senescence and subsequent capillary rarefaction in PO hearts remain largely unknown.

In this study, we found that hypertrophic cardiomyocytes paracrinally driven capillary rarefaction to promote PO-induced HF. Hypertrophic cardiomyocytes exerted this effect, at least in part, by secreting elevated levels of 3-methyl-2-oxovaleric acid (KMV), which paracrinally induced EC senescence and downregulated angiogenic factors by activating the transcription factor (TF) mesenchyme homeobox 2 (Meox2). The findings identified KMV as a novel metabolite secreted by hypertrophic cardiomyocytes that triggered EC senescence to drive PO-induced capillary rarefaction and subsequent HF. Targeting KMV-mediated hypertrophic cardiomyocyte-to-EC signaling may represent a promising therapeutic strategy for PO-induced HF via maintenance of an appropriate capillary density.

## Results

### Cardiac capillary density is negatively related to cardiomyocyte hypertrophy in PO-induced failing hearts

**Figure 1:**
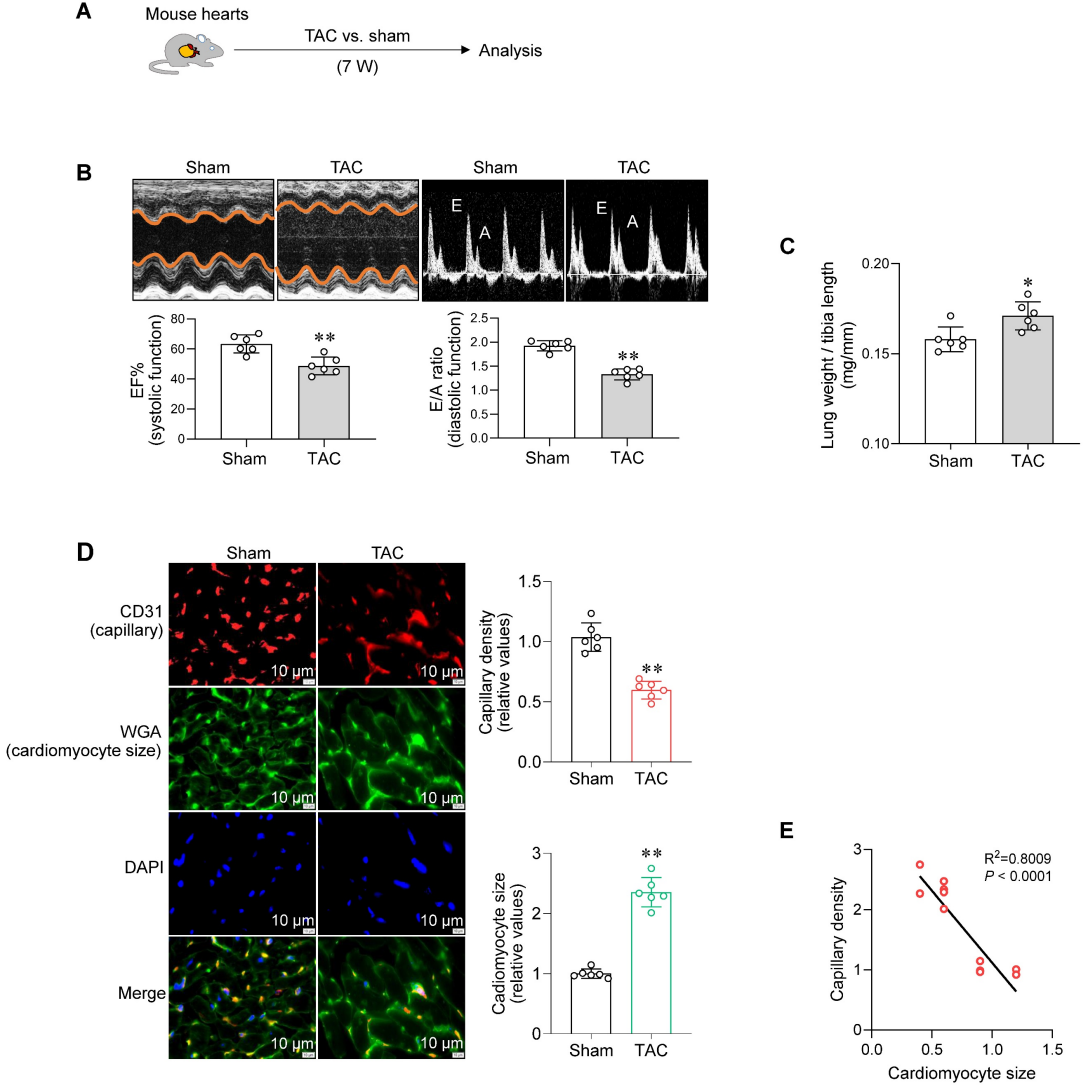
Cardiac capillary density was negatively related to cardiomyocyte hypertrophy in PO-induced failing hearts. A. Experimental diagram. Mice underwent TAC or sham surgeries for 7 weeks. Subsequently, the following analyses were performed. B. Cardiac function. Echocardiography was conducted to examine cardiac systolic (ejection fraction, EF%) and diastolic function (E wave to A wave ratio, E/A ratio). Data are mean ± SD, ** P < 0.01 by Student's two-tailed unpaired* t-*test. n = 6/group. C. Lung weight. The lung weight was examined and expressed as the ratio to tibia length (mg/mm). Data are mean ± SD, * P < 0.05 by Student's two-tailed unpaired* t-*test. n = 6/group. D. Cardiac capillary density and cardiomyocyte size. Cardiac frozen sections were prepared for CD31 immunostaining (red) to indicate capillary density and for WGA staining (green) to indicate cardiomyocyte size. DAPI (blue) was used to stain nuclei. Scale bar = 10 µm. Data are mean ± SD, ** P < 0.01 by Student's two-tailed unpaired* t-* test. n = 6/group. E. Relationship between capillary density and cardiomyocyte size. Linear correlation between cardiomyocyte size and capillary density was analyzed by linear regression test. n = 12/group.

To establish a model of PO-induced HF, mice underwent transverse aortic constriction (TAC) for 7 weeks according to previous studies 16, 17 (**Figure 1A**). TAC mice developed HF, which was characterized by decreased cardiac systolic function (ejection fraction, EF%) and diastolic function (E/A ratio and E/e' ratio), enlarged left ventricular chamber, and increased lung weight (**Figure 1B-C and S1, Table S1**). Meanwhile, TAC-induced failing hearts exhibited a reduced capillary density and enlarged cardiomyocyte size (**Figure 1D**). Remarkably, capillary density was negatively correlated with cardiomyocyte size in hearts (**Figure 1E**). These data indicate a possible link between cardiac capillary rarefaction and cardiomyocyte hypertrophy during PO-induced HF.

### Hypertrophic cardiomyocytes paracrinally impair angiogenesis

**Figure 2:**
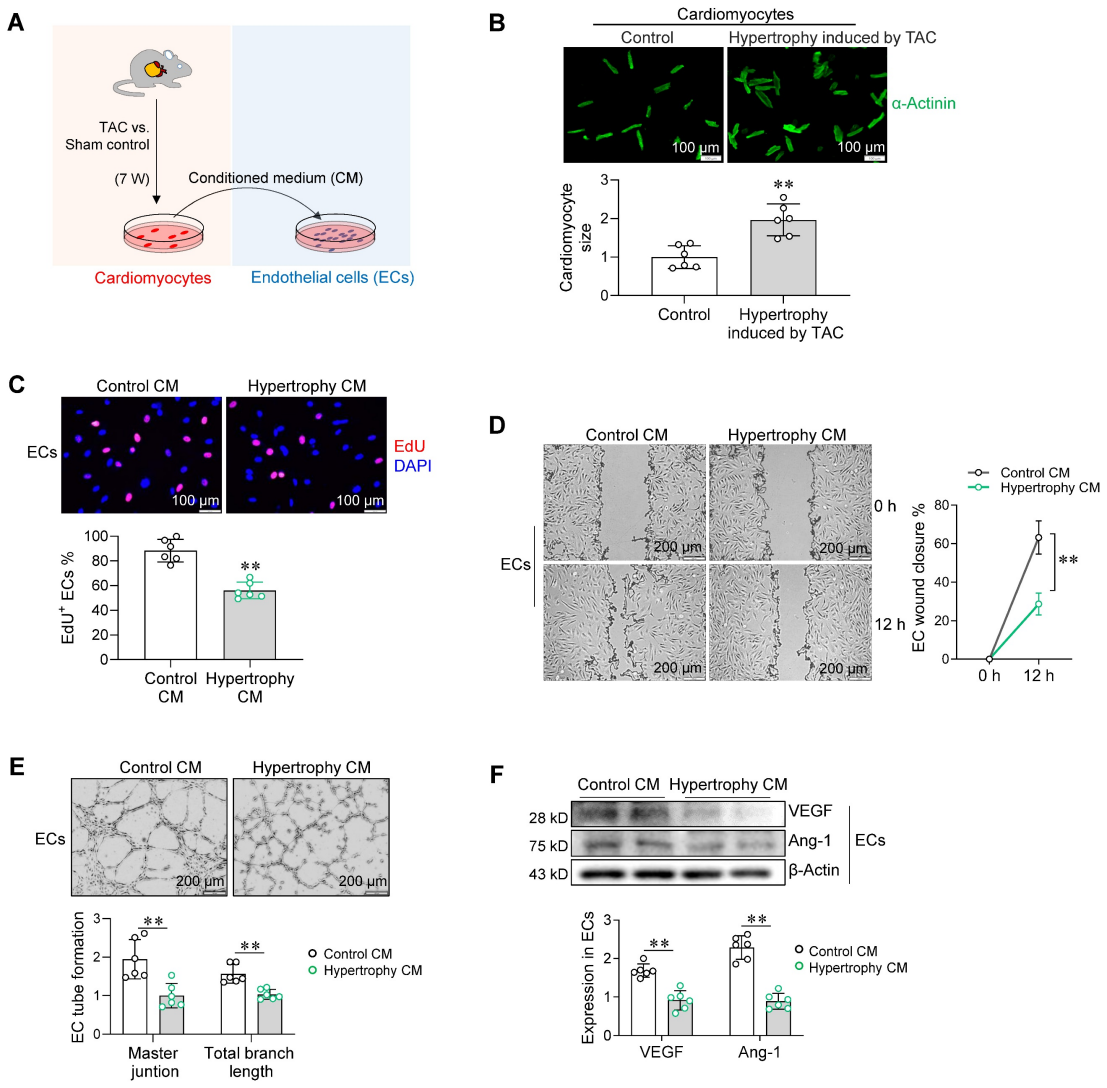
Hypertrophic cardiomyocytes paracrinally impaired angiogenesis. A. Experimental setting. Adult cardiomyocytes were isolated from hearts of mice after TAC or sham surgeries for 7 weeks. Following culture for 24 h, the medium of cardiomyocyte culture was collected as conditioned medium (CM) for EC treatment. B. Cardiomyocyte hypertrophy induced by TAC. Cardiomyocyte size was determined by immunostaining for α-Actinin. Scale bar = 100 μm. C-E. Effect of cardiomyocyte CM on endothelial angiogenesis. Following treatment with cardiomyocyte CM for 96 h, ECs were subjected to proliferation assay by EdU incorporation (C, Scale bar = 100 μm), migration assay by wound closure (D, Scale bar = 200 μm), and tube formation on Matrigel (E, Scale bar = 200 μm). F. Effect of cardiomyocyte CM on angiogenic factor expression in ECs. VEGF and Ang-1 expression were determined by immunostaining. Data are mean ± SD, ** P < 0.01 by Student's two-tailed unpaired* t-*test (B-C, E-F) and two-way ANOVA followed by Tukey's test (D). n = 6/group.

We then determined whether cardiomyocyte hypertrophy affects angiogenesis. To this end, primary cardiomyocytes were isolated from hearts of sham and TAC mice (**Figure 2A**). Cardiomyocytes from hearts of TAC mice exhibited hypertrophy (**Figure 2B**). Following culture for 24 h, the medium of cardiomyocyte cultures was collected as conditioned medium (CM) for treatment of primary ECs (**Figure 2A**). Notably, CM from hypertrophic cardiomyocyte cultures (hypertrophy CM) decreased EC proliferation in the EdU incorporation assay, delayed EC migration in the wound closure assay, and impaired EC tube formation on Matrigel assay when compared with control CM (**Figure 2C-E**). In support of these results, CM from hypertrophic cardiomyocyte cultures inhibited expression of the angiogenic factors vascular endothelial growth factor (VEGF) and angiopoietin-1 (Ang-1) in ECs (**Figure 2F**).

We also investigated the effect of *in vitro*-induced hypertrophic cardiomyocytes on angiogenic behavior of ECs. To this end, primary cardiomyocytes that isolated from neonatal rat hearts were treated with phenylephrine (PE) to induce hypertrophy according to previous studies (**Figure S2A-B**) 18. Similar to CM from cultures of TAC heart-derived hypertrophic cardiomyocytes, CM from PE-induced hypertrophic cardiomyocyte cultures inhibited EC proliferation, delayed EC wound healing, decreased EC tube formation, and downregulated VEGF and Ang-1 expression (**Figure S2C-F**). These data suggest that hypertrophic cardiomyocytes paracrinally impair angiogenesis.

### Hypertrophic cardiomyocytes secrete elevated levels of KMV

**Figure 3:**
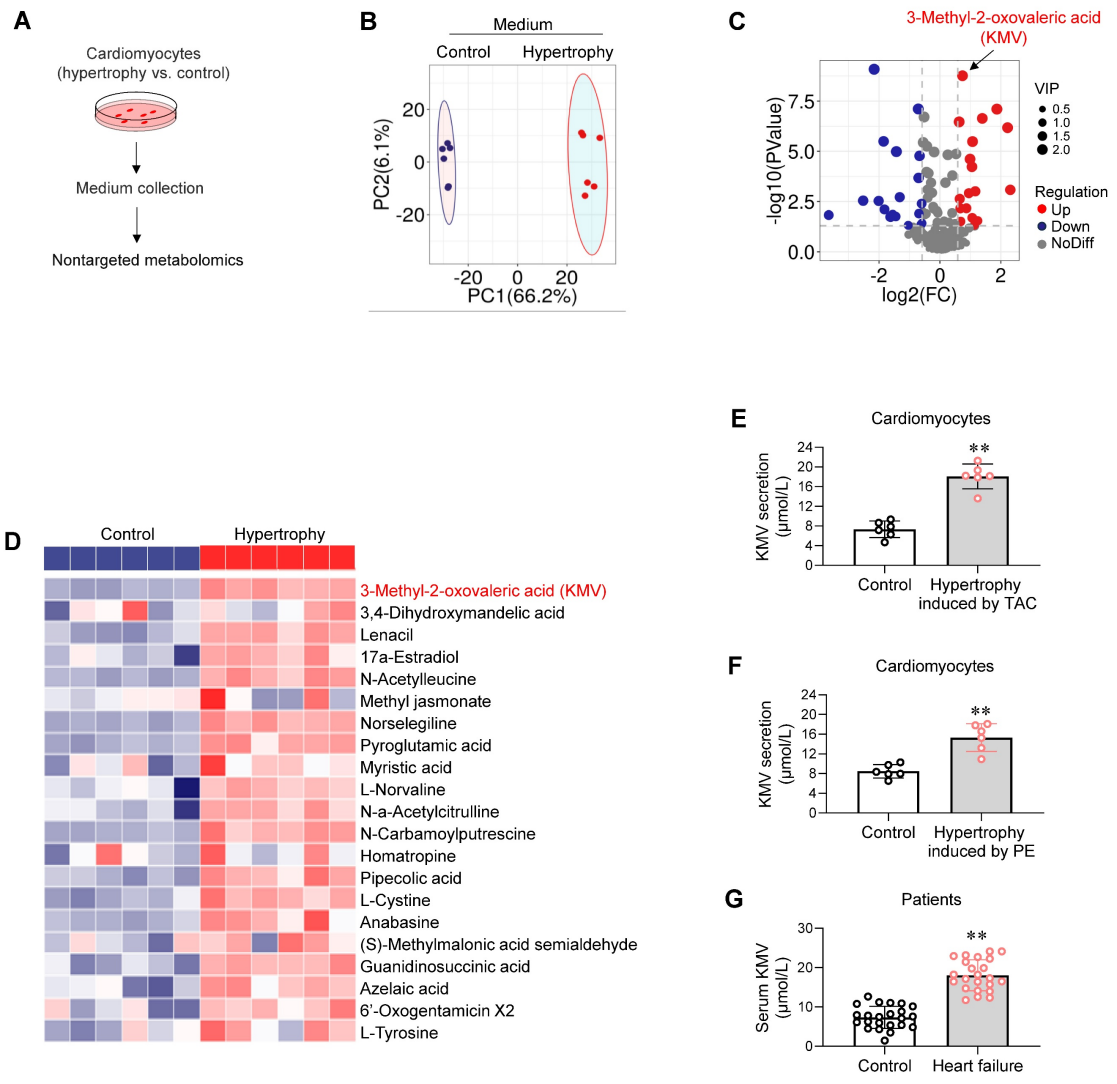
A-D. Hypertrophic cardiomyocytes secreted elevated levels of KMV. A-D. Untargeted metabolomics. The medium of cardiomyocyte cultures was collected for untargeted metabolomics (A). PCA plot showed separation of metabolite components between groups (B). Volcano plot showed upregulated, downregulated, and unchanged secreted metabolites (C). Heatmap showed the upregulated metabolites secreted by hypertrophic cardiomyocytes (D). E-G. ELISA analysis. ELISA was performed to compare KMV levels between the indicated groups. Data are mean ± SD, ** P < 0.01 by Student's two-tailed unpaired* t-*test. n = 6/group (E, F) and n = 23/group (G).

Metabolic alterations are found in hypertrophic and failing hearts 19, 20. To investigate whether hypertrophic cardiomyocytes secrete metabolites to impair angiogenesis, untargeted metabolomics was performed using culture medium of hypertrophic and control cardiomyocytes (**Figure 3A**). Principal component analysis showed a clear separation of metabolite secretion between the two groups (**Figure 3B**). Sixty-six secreted metabolites significantly differed between the two groups (adjusted *P <* 0.05, variable importance in projection > 1) (**Figure 3C**). Of these, KMV was the most upregulated secreted metabolite by hypertrophic cardiomyocytes (FDR = 1.39E-07, fold change = 1.670) (**Figure 3C-D**).

To verify this result, an enzyme-linked immunosorbent assay (ELISA) was performed to measure KMV secretion by cardiomyocytes into culture medium. Hypertrophic cardiomyocytes induced by TAC* in vivo* or by PE *in vitro* secreted higher levels of KMV than their controls (18.1 vs. 7.3 M or 15.3 vs. 8.5 M, respectively, **Figure 3E-F**). Serum of TAC mice showed higher KMV levels than sham controls (21.4 vs. 7.5 M, **Figure S3**). Additionally, serum of HF patients contained higher KMV levels than that of controls (19.3 vs. 5.2 M, **Figure 3G**).

### KMV impairs angiogenesis* in vitro*

**Figure 4:**
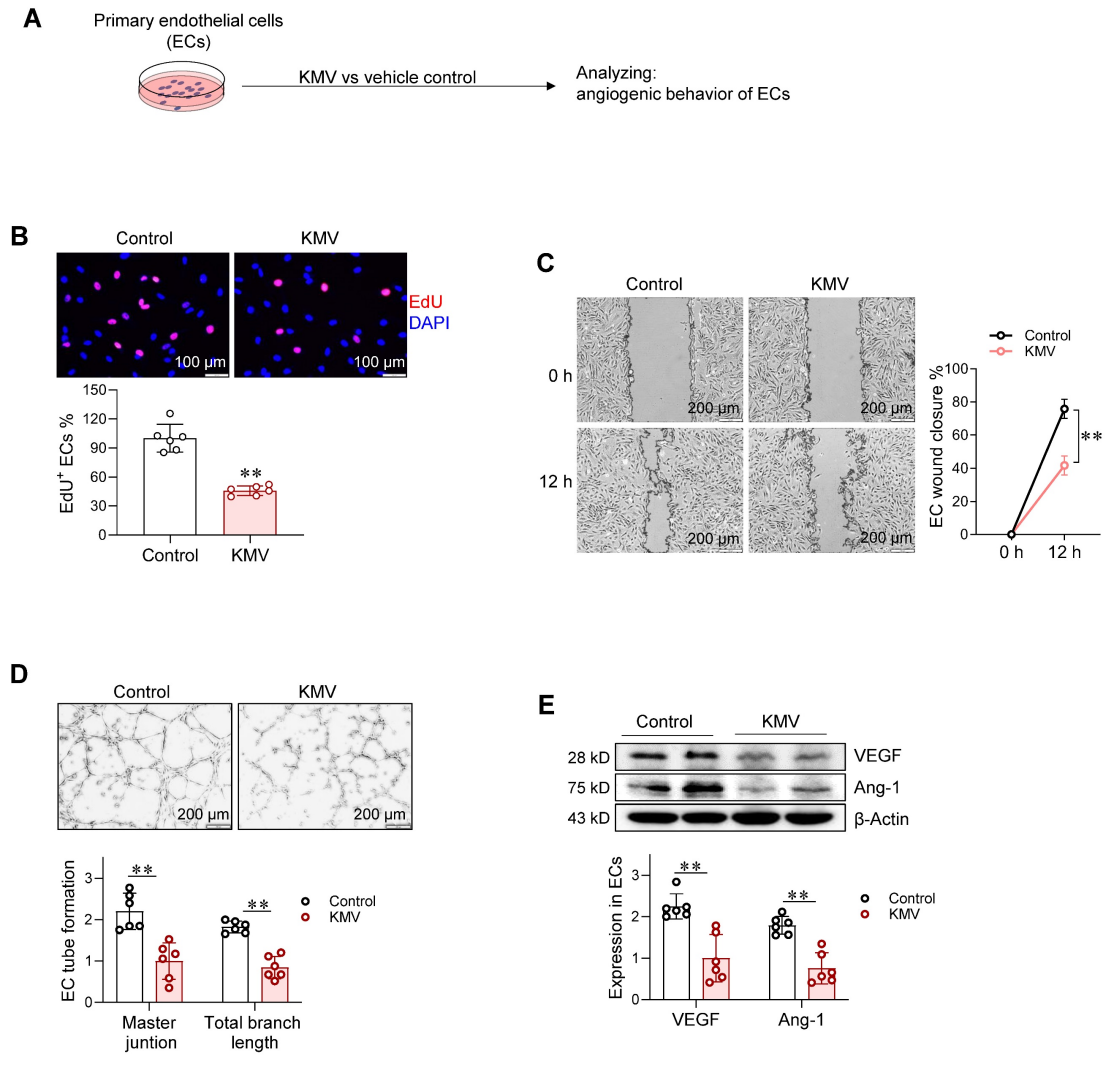
A. KMV impaired angiogenesis *in vitro*. Experimental setting. Following treatment with KMV or vehicle control for 96 h, ECs were subjected to the following analyses. B. EC proliferation. EdU incorporation assay was performed to examine EC proliferation. Scale bar = 100 μm. C. EC migration. EC migratory ability was examined by wound healing assay and expressed as the percentage of wound closure. Scale bar = 200 μm. D. Tube formation. The tube formation of ECs was examined by growing cells on Matrigel. Scale bar = 200 μm. E. Expression of VEGF and Ang-1. The indicated protein expression was examined by immunoblotting. Data are mean ± SD, ** P < 0.01 by Student's two-tailed unpaired* t-*test (B, D-E) and two-way ANOVA followed by Tukey's test (C). n = 6/group.

To evaluate the potential effect of KMV on angiogenesis, we conducted a two-sample Mendelian randomization analysis (https://www.ebi.ac.uk/gwas/). This analysis showed a potential correlation of KMV with cardiovascular disorders (**Figure S4A-B**). This motivated us to investigate whether KMV mediates the hypertrophic cardiomyocyte-induced impairment of angiogenesis. To this end, ECs were treated with KMV for 96 h at a concentration of 20 μg/mL (**Figure 4A**), which is a clinically relevant dose in serum of HF patients as shown in **Figure 3G**. Notably, KMV reduced EdU incorporation, wound closure, and tube formation of ECs compared with controls (**Figure 4B-D**). Moreover, VEGF and Ang-1 expression was lower in KMV-treated ECs than in control ECs (**Figure 4E**). Similarly, ECs treated with serum of HF patients, which contained higher KMV levels, exhibited decreases of EdU incorporation, wound closure, tube formation, and VEGF and Ang-1 expression (**Figure S5A-E**). Studies have shown that VE-cadherin and phosphorylated VE-cadherin (p**-**VE-cadherin) are also associated with angiogenesis 21. Indeed, increased p**-**VE-cadherin and decreased VE-cadherin levels were found in ECs following treatment with KMV, hypertrophy CM, or serum of HF patients (**Figure S6A-D**). These data suggest that KMV impairs angiogenesis *in vitro*.

In addition to KMV, we also screened the potential involvement of other upregulated metabolites in angiogenic impairment. Among the top five upregulated metabolites released from hypertrophic cardiomyocytes, KMV exhibited the highest increase, followed by: (1) 3,4-dihydroxymandelic acid (DHMA), an intermediate metabolite of noradrenaline predominantly produced in the heart 1; (2) Lenacil, an herbicide; (3) 17α-Estradiol, a hormone primarily generated by ovarian granulosa cells 2; and (4) N-Acetylleucine, a laboratory-synthesized substance 3. Given its physiological relevance, DHMA was selected to examine its effects on angiogenesis. To determine an optimal dosage of DHMA for the experiments, the dose-effect of DHMA on EC proliferation was examined. ECs were treated with DHMA for 24 h at concentrations of 10, 20, 40, 80, and 160 µM, based on previous studies 4. The MTT assay revealed that DHMA at these concentrations had no effects on EC viability (**Figure S7A**). Subsequently, 80 µM of DHMA was selected to investigate the time-effect of DHMA on EC proliferation. The MTT assay demonstrated that treatment with DHMA (80 µM) for 24, 48, and 96 h did not alter EC viability compared to untreated controls (**Figure S7B**). Notably, treatment with DHMA (80 µM) for 96 h also did not affect EdU incorporation, migratory ability, tube formation, or the expression of VEGF and Ang-1 in ECs (**Figure S7C-F**), suggesting that DHMA has no influence on angiogenesis.

### KMV aggravates PO-induced capillary rarefaction *in vivo*

**Figure 5:**
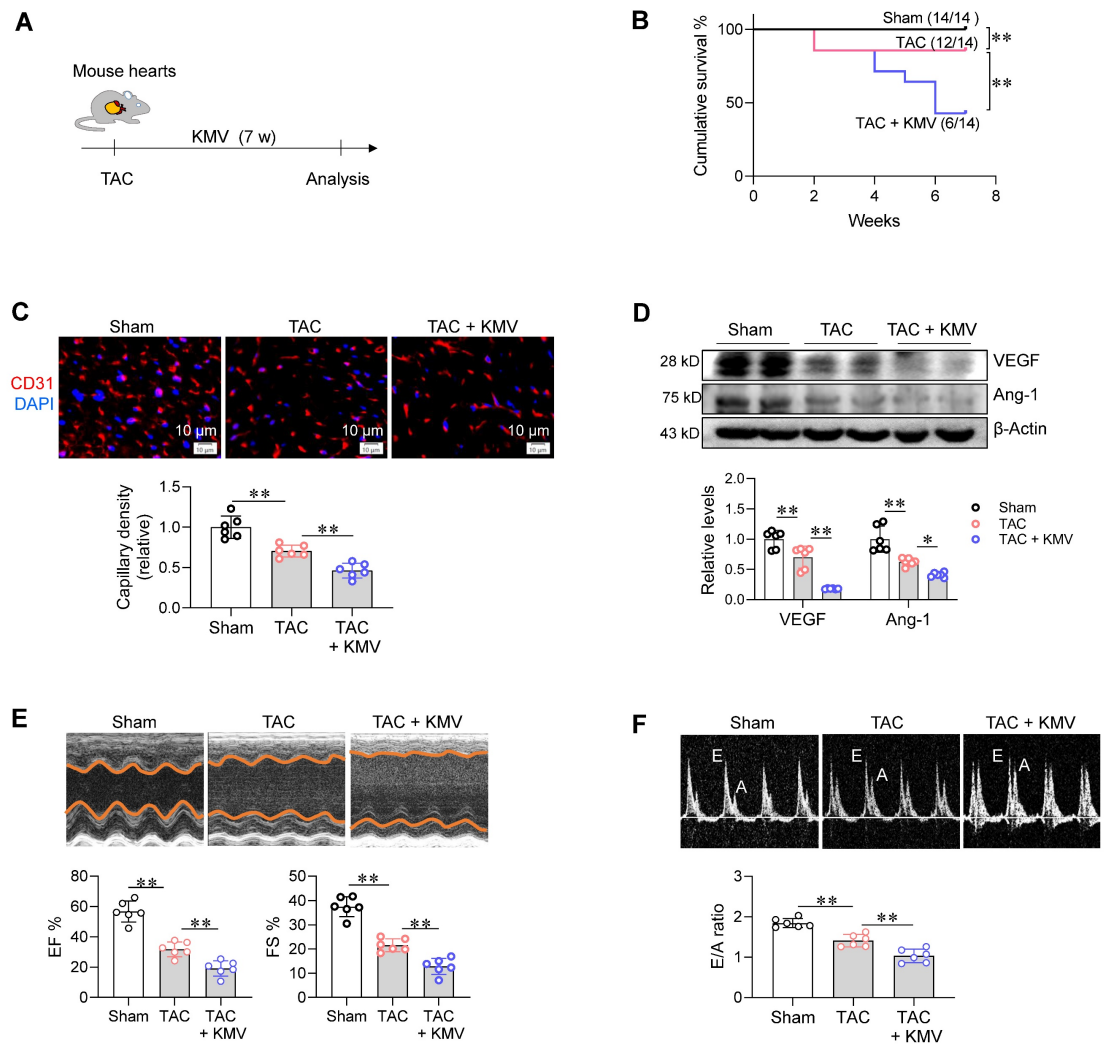
KMV aggravated PO-induced capillary rarefaction and cardiac dysfunction. A. Experimental diagram. Mice were assigned to three groups: sham surgery, TAC surgery, and TAC surgery + KMV treatment. B. Cumulative survival of mice. The survival of mice was recorded within 14 weeks after treatment. n = 14/group. C. Capillary density. After 7 weeks of treatment, heart tissues were prepared for frozen sectioning. The sections were stained with CD31 (red). DAPI was used to counterstain nuclei (blue). Scale bar = 10 µm. n = 6/group. D. VEGF and Ang-1 expression. After treatment for 7 weeks, heart tissues were prepared for immunoblotting against the indicated proteins. n = 6/group. E-F. Cardiac function. After treatment for 7 weeks, echocardiography was performed to examine cardiac systolic function (EF% and FS%) and diastolic function (E/A ratio). n = 6/group. Data are mean ± SD, * *P* < 0.05 and ** *P* < 0.01 by one-way ANOVA followed by Tukey's test (C-F) or by log-rank test (B).

To further investigate the role of KMV in PO-induced capillary rarefaction and the subsequent HF, TAC mice were treated with KMV (**Figure 5A**). Mice received KMV (500 μg/kg/day, intraperitoneally) based on previous studies 22, 23, and this treatment resulted in increased KMV levels in mouse serum compared to the non-treatment group (18.9 vs. 7.5 M, **Figure S3**). TAC decreased survival of mice, and this decrease was further enhanced by KMV treatment (**Figure 5B**). Notably, KMV treatment enhanced the TAC-induced decrease in capillary density in the heart (**Figure 5C**). In support of this, KMV treatment also enhanced the TAC-induced downregulation of VEGF and Ang-1 expression (**Figure 5D**). In addition, immunoblotting and immunostaining showed that the TAC-induced decrease in cardiac endothelial VEGFR2 expression was enhanced by KMV (**Figure S8A-B**). Furthermore, cardiac microvascular blood flow was evaluated using FITC-labeled *Lycopersicon esculentum* (Tomato) lectin, following the method described previously 10, 11. KMV enhanced the TAC-induced reduction in cardiac microvascular blood flow in mice (**Figure S9**). Moreover, echocardiographic examination showed that KMV treatment exaggerated the TAC-induced cardiac dysfunction, as reflected by decreases of EF%, fraction shortening (FS%), E/A ratio, E/e' ratio, and enlargement of left chamber (**Figure 5E-F and S10, Table S1**). These findings indicate that KMV aggravates capillary rarefaction and HF in mice following PO.

Additionally, we examined the effects of KMV on angiogenesis in mice without TAC. KMV decreased capillary density in the heart, vessel density in the retina, CD31^+^ glomerular area in the kidney, and CD31^+^ stained cells in the lung (**Figure S11A-D**). Supporting these findings, KMV inhibited VEGF and Ang-1 expression levels in these tissues (**Figure S12A-D**). Moreover, KMV increased cardiomyocyte size, ANP expression, and Collagen III expression in the hearts of mice without TAC (**Figure S13A-D**). These findings indicate that KMV has a wide spectrum of deleterious effects.

### KMV increases expression and nuclear accumulation of Meox2 in ECs

**Figure 6:**
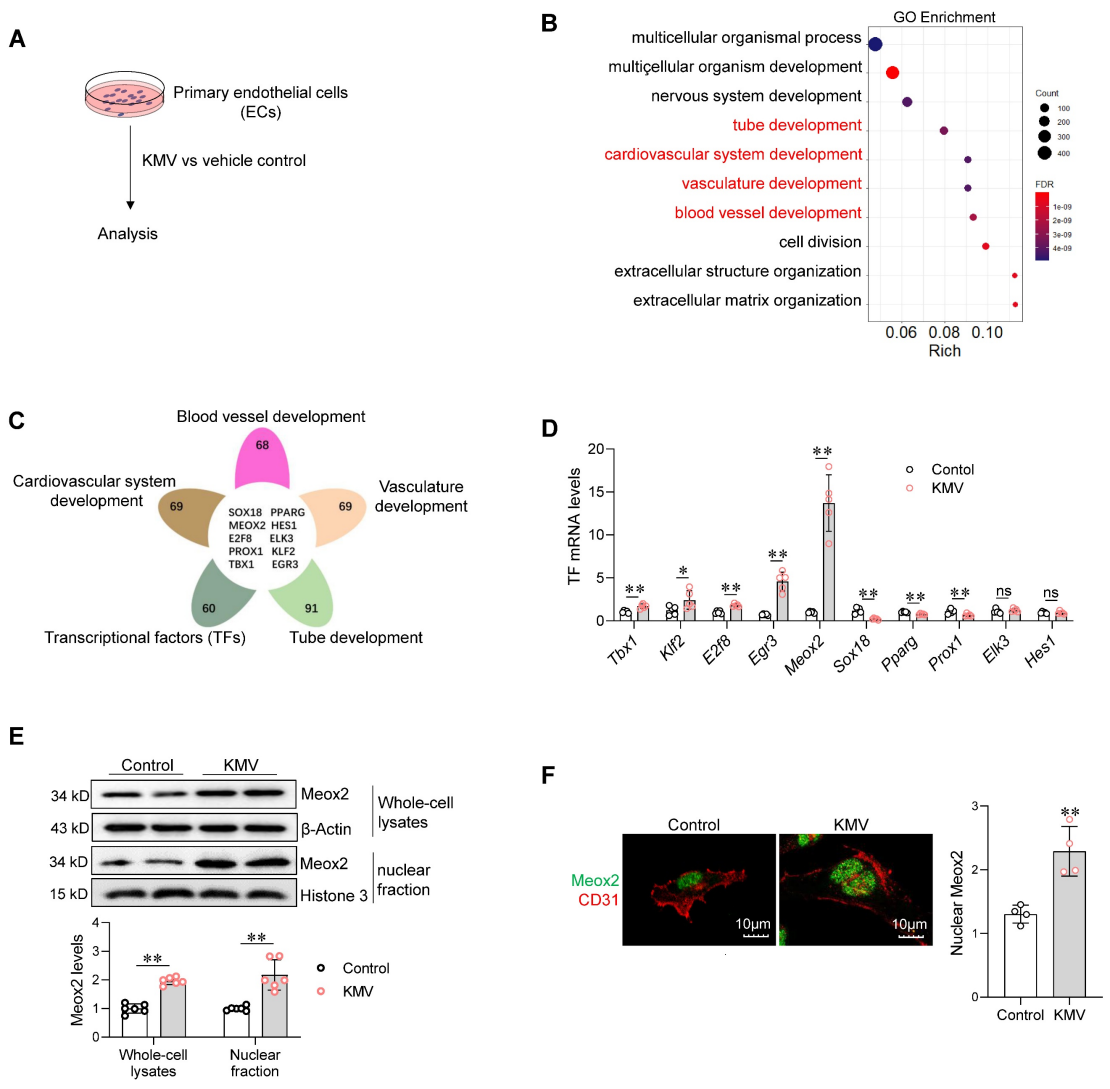
KMV increased expression and nuclear accumulation of Meox2 in ECs. A. Experimental setting. After treatment with KMV or vehicle control for 96 h, primary ECs were subjected to the following analyses. B-C. RNA-Seq. RNA-seq was performed in ECs. Go enrichment analysis of the differential expression genes (DEGs) between two groups (B). Venn diagram illustrates the integrative analysis of angiogenesis-related DEGs with the Animal Transcription Factor DataBase (C). D. qPCR. Expression of the indicated transcriptional factors was evaluated using qPCR. n = 5/group. ns, no significance. E. Meox2 protein expression. Meox2 expression was examined in whole-cell lysates and nuclear fractions of ECs using immunoblotting. n = 6/group. F. Meox2 distribution. Meox2 distribution was examined using immunostaining against Meox2 (green). CD31 (red) was used as an EC marker, and DAPI staining (blue) was used to label nuclei. n = 4/group. Scale bar = 10 µm. Data are mean ± SD, * *P* < 0.05, ** *P* < 0.01 by Student's two-tailed unpaired *t-*test.

To dissect the mechanisms underlying the deleterious effect of KMV on angiogenesis, RNA-sequencing (RNA-seq) was performed in ECs (**Figure 6A**). In KMV-treated ECs, downregulated differentially expressed genes (DEGs) were enriched in angiogenesis-related biological processes, including tube formation, cardiovascular system development, vasculature development, and blood vessel development (**Figure 6B**). By performing integrative analysis of these angiogenesis-related DEGs with the Animal Transcription Factor DataBase (https://guolab.wchscu.cn/AnimalTFDB#!/), we screened out ten TFs (**Figure 6C**). Among these ten TFs, quantitative PCR (q-PCR) revealed that mRNA expression of five were upregulated (*Tbx1, Klf2, E2f8, Egr3,* and* Meox2*), three were downregulated (*Sox18*, *Pparg*, and* Prox1*), and two were unchanged (*Elk3* and *Hes1*) in ECs treated with KMV (**Figure 6D**). Notably, *Meox2* was the most upregulated TF; thus, we focused on investigating its role in subsequent experiments. Immunoblotting revealed that the Meox2 protein content was higher in both whole-cell lysates and nuclear fractions of KMV-treated ECs than in those of control ECs (**Figure 6E**). Immunostaining confirmed that KMV increased nuclear accumulation of Meox2 (**Figure 6F**). Similar results were observed in mouse hearts, which demonstrated higher Meox2 expression in ECs (CD31^+^) of TAC hearts (**Figure S14A-C**).

### Meox2 mediates the KMV-induced impairment of angiogenesis

**Figure 7:**
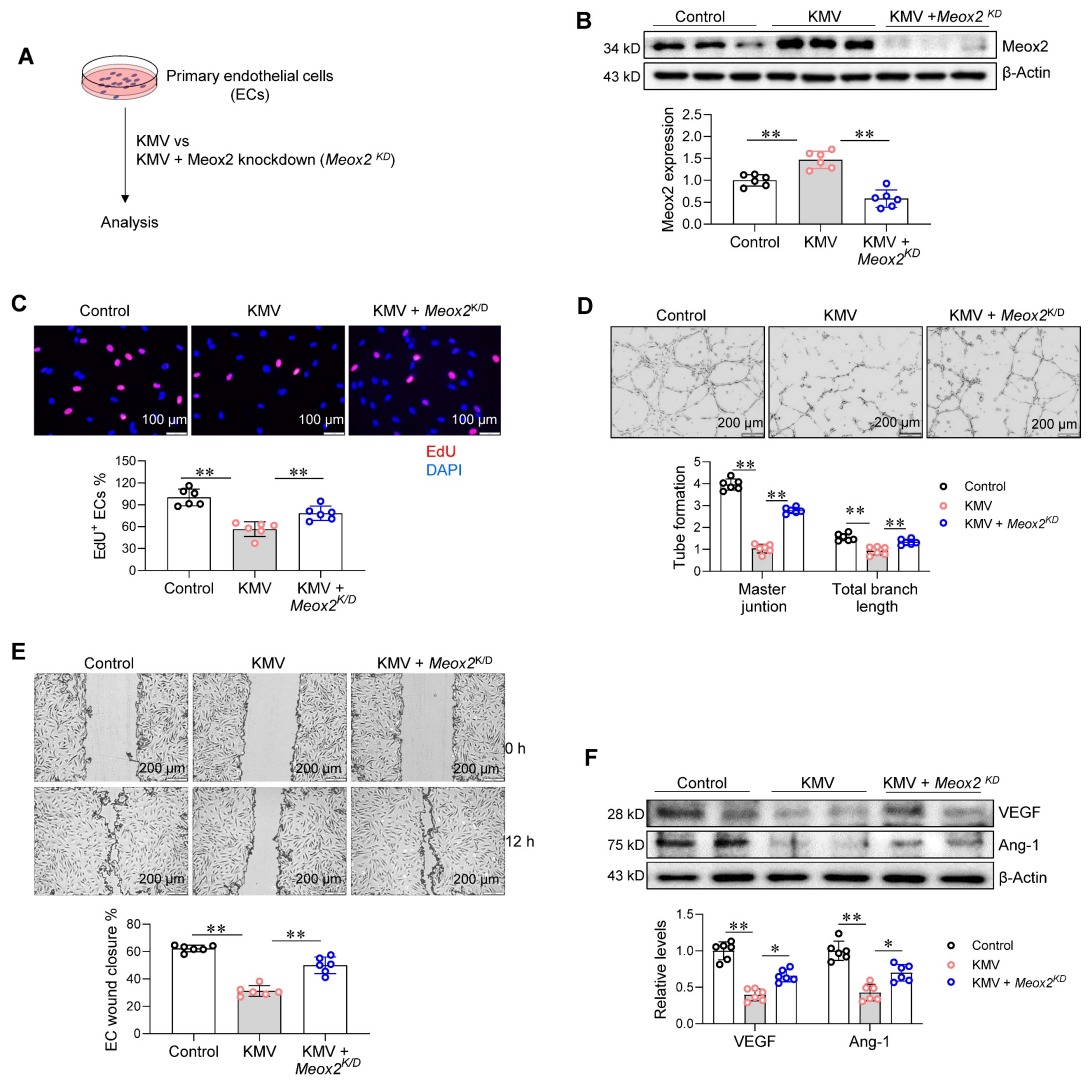
Meox2 mediated the KMV-induced impairment of EC angiogenesis. A. Experimental diagrams. Primary ECs were assigned to three groups: vehicle control, KMV, KMV + Meox2 knockdown (*Meox2^KD^*). After treatment for 96 h. The following experiments were performed subsequently. B. Meox2 knockdown. Knockdown of Meox2 was confirmed using immunoblotting. C. EC proliferation. EC proliferation was examined by EdU incorporation and expressed as percentage of EdU^+^ ECs over total ECs. Scale bar = 100 µm. D. Tube formation. Tube formation was examined by growth on Matrigel. Scale bar = 200 µm. E. EC migration. EC migratory ability was examined by wound healing assay and expressed as the percentage of wound closure. Scale bar = 200 μm. F. VEGF and Ang-1 expression. Immunoblotting was performed to examine VEGF and Ang-1 expression. Data are mean ± SD, * *P* < 0.05, ** *P* < 0.01 by one-way ANOVA followed by Tukey's test. n = 6/group.

The increased expression and nuclear accumulation of Meox2 prompted us to investigate whether this TF plays a role in KMV-induced impairment of angiogenesis. To this end, Meox2 was knocked down (*Meox2^KD^*) in primary ECs by transfecting *Meox2-*targeted siRNA (**Figure 7A**). The knockdown of Meox2 expression was confirmed by immunoblotting (**Figure 7B**). Notably, knockdown of Meox2 attenuated the KMV-induced decreases in EC proliferation (EdU incorporation), tube formation (Matrigel assay), and wound closure (**Figure 7C-E**). In support of these findings, Meox2 knockdown also attenuated the KMV-induced downregulation of VEGF and Ang-1 expression in ECs (**Figure 7F**). These data suggest that KMV impairs EC angiogenesis by activating Meox2.

### KMV induces EC senescence in a Meox2-dependent manner

**Figure 8:**
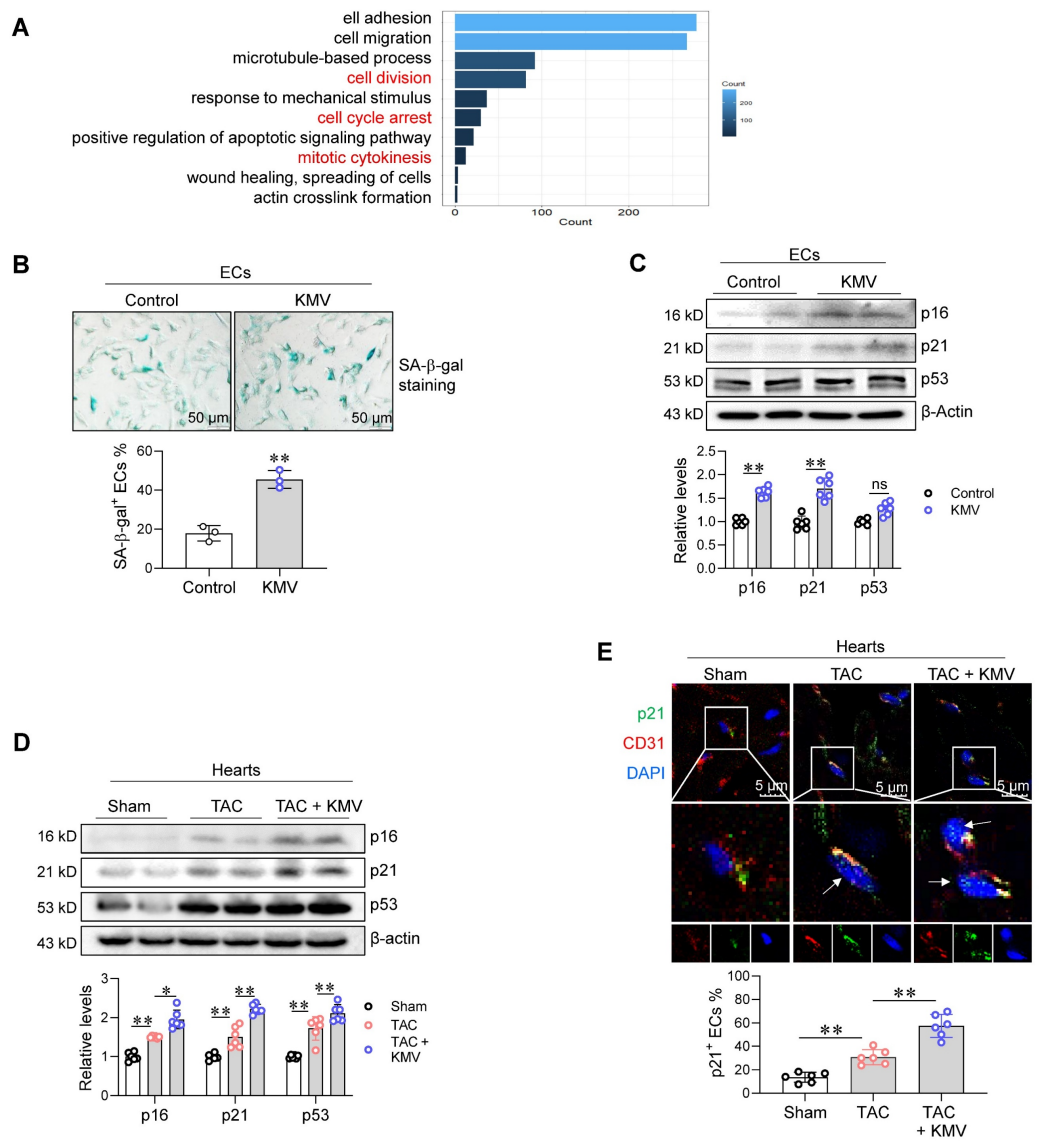
KMV induced EC senescence. A. KEGG diagram. RNA-Seq data showed an enrichment of senescence-related biological processes in KMV-treated ECs. B-C. Effect of KMV on EC senescence *in vitro*. Following KMV treatment for 96 h, SA-β-gal staining was performed and expressed as the percentage of SA-β-gal^+^ ECs over total ECs (B, scale bar = 50 μm, n = 3/group). Expression of p16, p21, and p53 was examined by immunoblotting (C, n = 6/group). Data are mean ± SD, ** *P* < 0.01 by Student's two-tailed unpaired *t-*test. ns, no significance. D-E. Effect of KMV on EC senescence in TAC heart *in vivo*. Following treatment for 7 weeks, hearts were prepared for immunoblotting against the indicated proteins (D). Also, immunostaining against p21 (green) and CD31 (red) was performed on frozen heart sections, and arrows indicate the p21^+^ ECs in hearts (E, scale bar = 5 μm). Data are mean ± SD, * *P* < 0.05, ** *P* < 0.01 by one-way ANOVA followed by Tukey's test, n = 6/group.

Previous studies have reported that Meox2 plays a role in EC senescence 24, 25. Considering that EC senescence impairs angiogenesis 9, 10, we investigated whether EC senescence is involved in KMV-induced angiogenesis impairment. To this end, we re-analyzed our RNA-seq data. This analysis showed that DEGs in KMV-treated ECs were also enriched in senescence-related biological processes, including cell division, cell cycle arrest, and mitotic cytokinesis (**Figure 8A**). Indeed, KMV increased the percentage of SA--gal^+^ ECs and upregulated the expression of the senescence markers p16 and p21 in ECs (**Figure 8B-C**). Similar results were obtained in ECs treated with CM of hypertrophic cardiomyocytes, which displayed increased SA--gal^+^ staining and higher p16 and p21 expression (**Figure S15A-C**). Importantly, TAC increased p16 and p21 expression in the hearts of mice, and this increase was augmented by KMV treatment (**Figure 8D**). Moreover, immunostaining revealed that KMV enhanced the TAC-induced upregulation of p21 in ECs of mouse hearts (**Figure 8E**).

**Figure 9:**
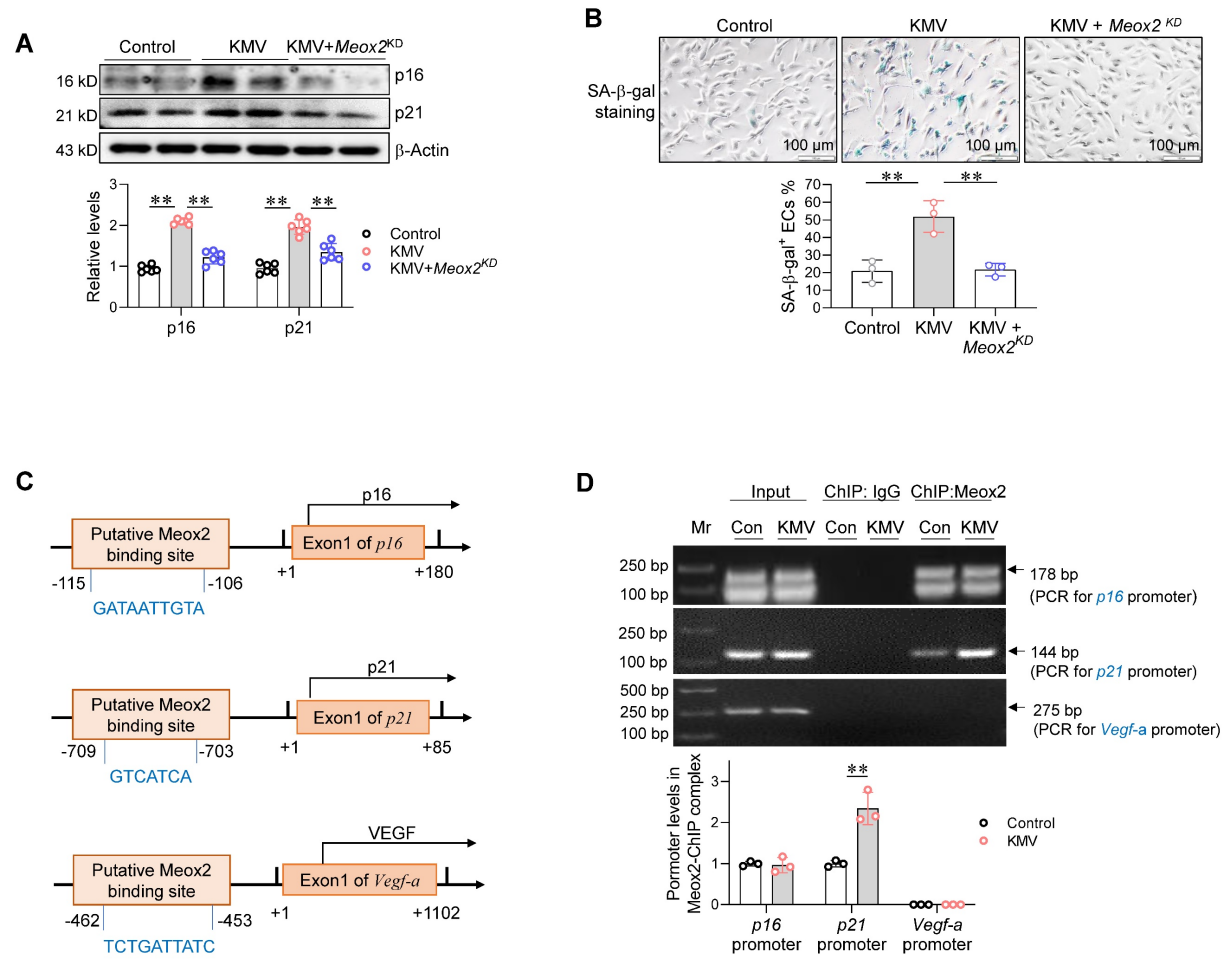
Meox2 mediated KMV-induced endothelial senescence. A-B. Effects of Meox2 knockdown on KMV-induced EC senescence. Primary ECs were assigned to three groups: vehicle control, KMV, KMV + Meox2 knockdown (*Meox2^KD^*). After treatment for 96 h, ECs were subjected to immunoblotting against p16 and p21 (A, n = 6/group) and SA-β-gal staining (B, scale bar = 100 μm, n = 3/group). Data are mean ± SD, ** *P* < 0.01 by one-way ANOVA followed by Tukey's test. C. The predicted Meox2 binding sites in promotor regions of the indicated genes. D. ChIP-PCR. Primary ECs were treated with vehicle control or KMV for 96 h. ChIP was performed against Meox2. The Meox2 ChIP complex was used for PCR to amplify the predicted promoter regions. Data are mean ± SD, ** P < 0.01 by Student's two-tailed unpaired *t*-test. n = 3/group.

Next, we examined the effect of Meox2 on KMV-induced EC senescence. Intriguingly, knockdown of Meox2 diminished the KMV-induced increases of p16 and p21 expression and SA--gal^+^ staining in ECs (**Figure 9A-B**). These results suggest that KMV induces EC senescence in a Meox2-dependent manner.

Finally, we sought to determine whether Meox2 acts as a TF to directly drive expression of genes related to senescence and angiogenesis, such as *p16*, *p21*, and *Vegf*. Gene promoter analysis (http://bioinfo.life.hust.edu.cn/hTFtarget#!/) showed that a putative binding site for Meox2 is present in the* p16* promoter at position -115/-106, in the *p21* promoter at position -709/-703, and in the *Vegf-a* promoter at position -462/-453 (**Figure 9C**). To determine whether Meox2 binds to these promoter regions, a chromatin immunoprecipitation (ChIP) assay was performed using an anti-Meox2 antibody. PCR using this ChIP complex showed that Meox2 bound to the promoter regions of *p16* and* p21* but not to that of *Vegf-a*. Notably, KMV increased Meox2-*p21* promoter binding in ECs (**Figure 9D**).

In addition to Meox2, KMV also increased the mRNA levels of four other TFs, including *Egr3, Klf2, Tbx1, and E2f8*, in ECs (**Figure 6D**). However, immunoblotting showed that KMV upregulated the protein level of TBX1 but did not affect the protein levels of KLF2, Egr3, or E2F8 in ECs (**Figure S16A**). To determine the potential involvement of TBX1 in KMV-induced EC senescence, TBX1 was knocked down (**Figure S16B**). TBX1 knockdown did not significantly alter the KMV-induced increases in SA-β-gal staining or the expression of p16 and p21 in ECs (**Figure S16C-D**).

## Discussion

**Figure 10:**
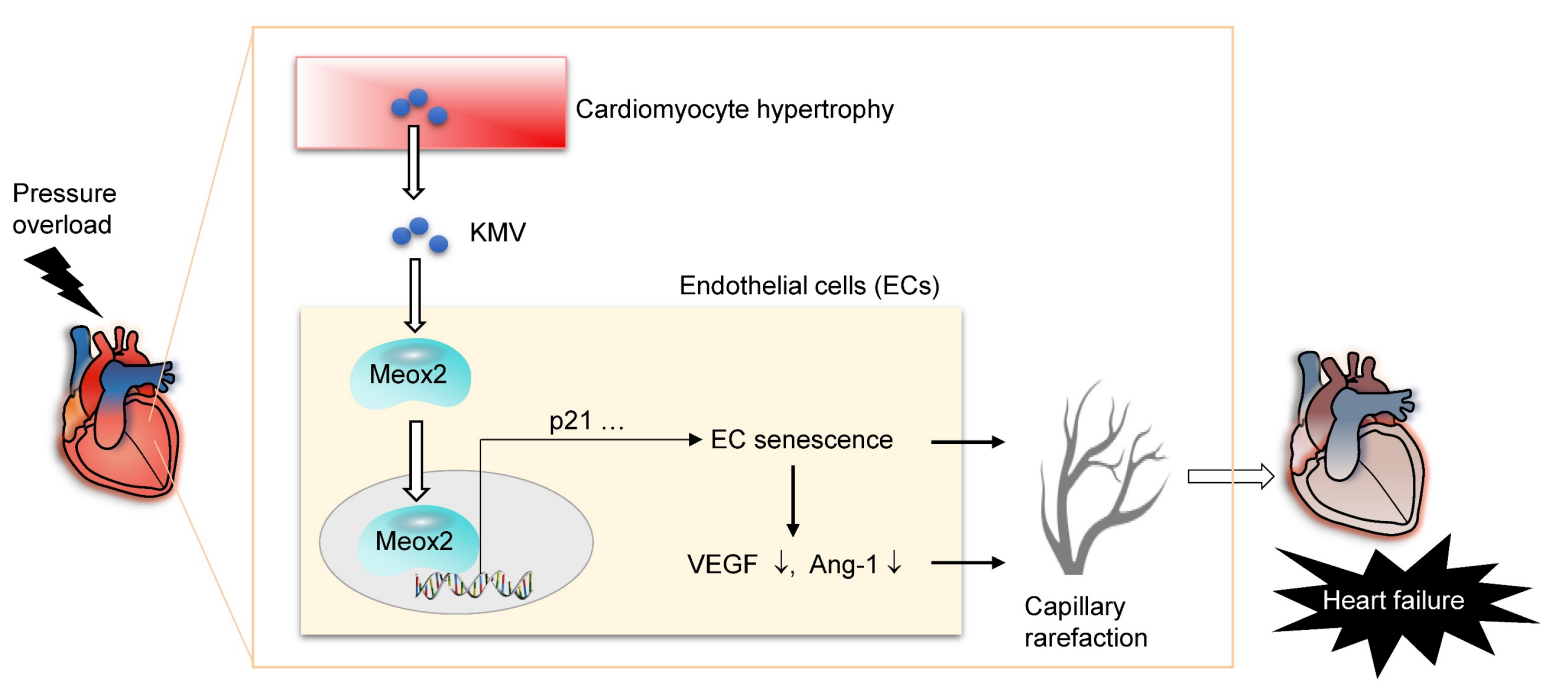
Mechanistic scheme. In pressure overload (PO) hearts, hypertrophic cardiomyocytes paracrinally drive cardiac capillary rarefaction to promote heart failure (HF) development. This action of hypertrophic cardiomyocytes is mediated, at least in part, through secreting elevated levels of KMV, which paracrinally increases expression and nuclear accumulation of Meox2 in ECs, where Meox2 binds to the promoter of *p21*, thereby triggers EC senescence and subsequent angiogenesis impairment, ultimately drives capillary rarefaction to promote development of PO-induced HF.

In this study, we sought to elucidate the role of cardiomyocyte-to-EC signaling in the development of capillary rarefaction in the context of PO-induced HF. The findings identified KMV as a metabolite secreted by hypertrophic cardiomyocytes that paracrinally triggered EC senescence to drive PO-induced capillary rarefaction and subsequent HF development (**Figure 10**). Targeting the KMV-mediated cardiomyocyte-to-EC signaling represents a potential therapeutic intervention for patients with PO-induced HF.

Converging evidence from basic research and clinical studies has consistently demonstrated that capillary density is significantly reduced in failing hearts, and the occurrence of capillary rarefaction coupled with impaired angiogenesis is considered a hallmark of PO-induced HF 26, 27. The diffuse capillary loss (capillary) throughout the myocardium results in inadequate delivery of oxygen and nutrients to the heart. PO induces cardiomyocyte hypertrophy, and hypertrophic cardiomyocytes have a higher demand for energy metabolism; therefore, inadequate delivery of oxygen and nutrients ultimately drives decompensated ventricular dilation and cardiac dysfunction. Thus, the mismatch between cardiac high energy demand and vascular rarefaction is a critical factor contributing to pressure overload-induced heart failure. In corroboration of these findings, our study revealed that TAC-induced failing hearts exhibited a reduced capillary density accompanied by cardiomyocyte hypertrophy. Moreover, we observed a negative correlation between cardiac capillary density and cardiomyocyte size, suggesting a potential mechanistic link between cardiac capillary rarefaction and cardiomyocyte hypertrophy in the pathogenesis of PO-induced HF.

The mammalian heart comprises a complex and heterogeneous cellular composition, with cardiomyocytes coexisting with a diverse population of non-cardiomyocytes, including ECs, fibroblasts, and macrophages 28. In response to pathological stimuli, such as PO, these diverse cardiac cell populations engage in intricate intercellular communication via paracrine and endocrine signaling pathways, which plays a pivotal role in orchestrating the onset and progression of HF 29, 30. Metabolic alterations, such as changes in substrate utilization, energy production, and metabolite accumulation and secretion, are not only a hallmark of but also a contributor to cardiac hypertrophy and HF development 19, 20. In this study, we employed a combination of untargeted metabolomics and targeted ELISA to identify the metabolites secreted by hypertrophic cardiomyocytes that may paracrinally influence the angiogenic behavior of ECs and subsequently capillary density. A key finding of our work is that KMV, a metabolic intermediate of isoleucine catabolism, is a novel metabolite secreted by hypertrophic cardiomyocyte that impairs the angiogenic behavior of ECs, leading to capillary rarefaction. KMV is one of the three branched-chain keto acids (BCKAs), which are metabolic derivatives of branched-chain amino acids (BCAAs). Although abnormal BCAA or BCKA metabolism is closely linked to various diseases 31, 32, the specific biological role of KMV, particularly in the context of PO-induced HF, remains poorly understood. Our findings expand the repertoire of metabolites that can influence angiogenic behavior of ECs and highlight the importance of cardiomyocyte-derived metabolites in cardiac capillary rarefaction for the development of PO-induced HF.

The induction of EC senescence by KMV is another significant finding of our study. EC senescence is a crucial driver of capillary rarefaction in various cardiovascular diseases, including HF 9, 10. Senescent ECs exhibit decreased angiogenic factor (e.g. VEGF and Ang-1) expression, impaired proliferative and angiogenic capacities, as well as increased secretion of inflammatory factors and extracellular matrix-degrading enzymes, all of which can lead to reduced capillary density and deterioration of the myocardial microenvironment 33. However, the factors and underlying molecular mechanisms that mediate EC senescence in PO hearts remain largely unknown. In this study, we made the following findings: (1) hypertrophic cardiomyocytes paracrinally triggered EC senescence; (2) KMV was the most unregulated metabolite secreted by hypertrophic cardiomyocytes, and KMV treatment induced EC senescence in cultured ECs *in vitro* as well as in TAC hearts *in vivo*; and (3) KMV exaggerated PO-induced capillary rarefaction and subsequent HF development. In summary, our study identified KMV-mediated hypertrophic cardiomyocyte-to-EC signaling that triggers EC senescence and, subsequently, capillary rarefaction to ultimately drive PO-induced HF.

Emerging evidence suggests that a diverse array of metabolites, derived from carbohydrate, lipid, and protein metabolism, have functions beyond their conventional roles in energy production and biosynthesis. These metabolites possess non-canonical functions, including roles in cell signaling cascades and regulation of gene expression 34. In light of these findings, we sought to elucidate the molecular mechanisms underlying KMV-induced EC senescence and subsequent capillary rarefaction. Through RNA-seq and molecular biology experiments, we identified Meox2 as a potential TF involved in KMV-induced impairment of angiogenesis. Evidence suggests that Meox2 functions as a potent inhibitor of angiogenesis in various pathological conditions, including neoplastic diseases and cardiovascular disorders 35, 36. Additionally, Meox2 has been implicated in the mediation of cell senescence. In this study, we made the following findings: (1) KMV increased the expression and nuclear accumulation of Meox2 in ECs; (2) knockdown of Meox2 attenuated KMV-induced EC senescence and angiogenesis impairment; and (3) Meox2 directly bound to the promoters of *p16* and *p21* but not to that of *Vegf-a*, and KMV enhanced the binding of Meox2 to the *p21* promoter. Collectively, these results suggest that Meox2 is an important TF that mediates the deleterious effect of KMV, which is secreted by hypertrophic cardiomyocytes, on EC senescence and capillary rarefaction in PO hearts.

In conclusion, this study identified KMV-dependent cardiomyocyte-to-EC signaling as a mechanism that triggers capillary rarefaction to drive PO-induced HF. These findings not only deepen our understanding of the pathogenesis of HF but also provide a potential therapeutic strategy for treatment of patients with HF.

## Materials and methods

### Reagents

The following materials were sourced from Sigma-Aldrich (St. Louis, MO): trypsin, paraformaldehyde (PFA), gelatin, and wheat germ agglutinin (WGA), 3-methyl-2-oxovaleric acid (KMV), Chromatin Immunoprecipitation (ChIP) Assay Kit, and MTT [3-(4, 5-Dimethylthiazol-2-yl)-2,5-diphenyltetrazolium bromide] reagent. Matrigel was obtained from Mogengel-Bio (Shanghai, China). DAPI was obtained from Cell Signaling Technology (Beverly, MA). Collagenase Type II was procured from Worthington Biochemical Corporation (Lakewood, NJ). Bovine serum albumin (BSA) was sourced from Roche (Basel, Switzerland). SA-β-gal staining kit and nuclear protein extraction kits were from Beyotime (Shanghai, China). M199 medium and fetal bovine serum (FBS) were procured from Gibco (Shelton, CT). Horse serum was obtained from Biological Industries (Kibbutz Beit Haemek, Israel). DHMA was from MedChemExpress (Monmouth Junction, NJ). FITC-labeled Lycopersicon esculentum (Tomato) lectin was from Vector Laboratories (Newark, CA). The high-sensitivity ECL Western blotting substrate was sourced from Tanon (Shanghai, China). The ELISA kit was purchased from RuiChuang Biotechnology Co., Ltd (Tianjin, China).

### Human serum samples

Human serum samples, which were used for measuring KMV in circulating blood, were collected from HF patients in the First Affiliated Hospital of Nanjing Medical University (Nanjing, China). Serum samples from non-HF patients served as controls. The HF group was included based on the following criteria: aged ≥18, New York Heart Association (NYHA) Class II or above, ejection fraction (EF%) 40%, and brain natriuretic peptide (BNP) > 150 ng/L. The non-HF control group included individuals aged ≥18, NYHA Class I, EF% > 55%, and BNP < 35 ng/L. Exclusion criteria included severe untreated heart valve disease, severe hepatic or renal insufficiency, prolonged prothrombin time, severe infections, and other conditions such as diabetes, obesity, chronic obstructive pulmonary disease, malignancy, chronic renal failure prior to the study, and ongoing corticosteroid therapy. This study was approved by the Ethical Board of First Affiliated Hospital of Nanjing Medical University (#2024-SR-664). Individuals gave informed consent at the time of recruitment. All the human studies were conducted in accordance with the principles outlined in the WMA Declaration of Helsinki and the Department of Health and Human Services Belmont Report.

### Animals

Mice (C57BL/6 background) were housed in Model Animal Research Center of Nanjing University and maintained in the Animal Laboratory Resource Facility at Nanjing University. All animal experiments conform to the guidelines from Directive 2010/63/EU of the European Parliament on the protection of animals used for scientific purposes. The animal care and experimental protocols were approved by the Nanjing University Committee on Animal Care (#GX55). All the experiments complied with the international guidelines on the ethical use of animals. Mice were randomly assigned to all analyses. Investigators were blinded to the echocardiographic measurements and histological analysis. Investigators were not blinded to animal handling, sampling, and raw data collection.

### Pressure overload

Pressure overload was induced by transverse aortic constriction (TAC) surgery in 8-week-old male mice according to the established standard procedures 16, 17. Mice were anesthetized with 2% isoflurane (R510-22-10, RWD Life Science, Shenzhen, China) and anaesthesia was monitored by the lack of reflex response to toe pinching. After anaesthesia, mice were mechanically ventilated, and aortic arch was accessed via a left lateral thoracotomy. Following placed a 27-gauge needle alongside the transverse aorta, a ligation was performed between the innominate and left carotid arteries using 5-0 suture. The needle was immediately removed after ligation, leaving the aortic arch constricted to the diameter of the needle. In sham-operated animals, the same procedure was performed except for the aorta ligation. Respiratory rate, body temperature, and heart rate were monitored throughout surgery. For analgesia, buprenorphine (0.05 mg/kg) was administrated subcutaneously prior to surgery, and every 8 h for the next 48 h, as described in our previous studies 37. For tissue collection, mice were euthanized by overdose anaesthesia (pentobarbital sodium 150mg/kg intraperitoneal injection) and cervical dislocation according to previous study 38.

### Cardiac function measurement

Cardiac function was examined using echocardiography according to our previous methods 38, 39. Briefly, mice were anesthetized by inhalation with 1-1.5% isoflurane. Images were taken by using the Vevo770 system equipped with a 35-MHz transducer (Visualsonics, Toronto, Canada). To analyze E/e' ratio, tissue Doppler imaging was conducted using Vevo3100 echocardiographic system (Vevo3100, Visualsonics, Canada). The examination was conducted by an observer who was blinded to the treatment. Parameters were obtained from M- mode, pulse Doppler tracings, and tissue Doppler imaging, and values were averaged using four to five cardiac cycles.

### Cardiomyocyte hypertrophy measurements

***In mouse heart.*** Following TAC or sham surgeries for 7 weeks, heart tissues at papillary levels were collected and prepared for frozen sectioning. The sections were stained with WGA (10 μg/mL) for 30 minutes to indicate cardiomyocytes size according to our previous methods 39. DAPI was used to stain nuclei.

***For primary cardiomyocytes.*** after fixation with 4% PFA, cardiomyocytes were immunostained against α-actinin overnight at 4° C followed by incubation with Alexa Fluor 488-conjugated secondary antibodies. DAPI was used to stain the nuclei. The staining was observed by using a fluorescence microscope and quantified by using Cellsens Dimension 1.15 software (Olympus, Tokyo, Japan).

### Cardiac capillary density

Following TAC or sham surgeries for 7 weeks, cardiac tissues were obtained at papillary muscle level and prepared for frozen sectioning. To evaluate cardiac capillary density, immunostaining against CD31 was performed according to our previous methods 40. DAPI was used to stain nuclei. The capillary density was quantified by using Cellsens Dimension 1.15 software (Olympus, Tokyo, Japan).

### Isolation and culture of primary cardiomyocytes and endothelial cells (ECs)

***Primary cardiomyocytes from adult mouse hearts.*** These cardiomyocytes were isolated from hearts of mice after TAC or sham surgery for 7 weeks using Langendorff perfusion system according to previous studies 41. The buffers and medium used are listed in the **Tables S2-S5**. A brief overview of the isolation and plating procedure for cardiomyocytes is shown in **Figure S17**. In brief, after mice were euthanized as mentioned above, hearts were rapidly removed, cannulated via ascending aorta. Then, hearts were retrogradely perfused with perfusion buffer, collagenase buffer, and calcium-containing collagenase buffer. Subsequently, the hearts were minced, triturate, and filtered (100 µm). Calcium was gradually reintroduced to the cell suspension to a final concentration of 1.0 mM. Finally, the cells were resuspended in plating medium and placed in a cell culture dish coated with laminin (15 µg/mL). One hour later, culture medium was changed to maintenance medium, and the medium was refreshed daily thereafter.

***Primary cardiomyocytes from neonatal rat hearts.*** These cardiomyocytes were isolated from hearts of neonatal rats (1-3 day-old) according to our previous methods 38, 42, 43. A brief overview of the isolation and plating procedure for cardiomyocytes is shown in **Figure S18**. Briefly, newborn Sprague-Dawley rats were euthanized by hypothermic anaesthesia and decapitation. The cardiac ventricular tissues were minced to a size of 1-2 m, digested with 0.04% collagenase type II and 0.06% trypsin, and centrifuged at 1000 rpm for 5 min. The pellets were resuspended in DMEM supplemented with 10% FBS. After pre-plating for 2 h, the unattached cells, as considered to be cardiomyocytes, were collected for plating on other dishes. The cardiomyocytes were cultured in DMEM containing 5% FBS and 10% horse serum.

***Primary ECs from human umbilical vein*.** Primary ECs were obtained from the umbilical vein cords of normal pregnancies according to our previous methods 44, 45. Briefly, ECs were dissociated from umbilical veins using 0.25% trypsin and grown in M199 medium supplemented with 10% FBS and 0.5 ng/mL bFGF. The ECs from passages 2 to 5 were used in the experiments. The studies were approved by the Ethical Board of the First Affiliated Hospital of Nanjing Medical University (#2021-SR-104). All human study procedures were followed in accordance with the ethical standards of the responsible committee on human experimentation and with the Helsinki Declaration of 1975, as revised in 2000.

### Treatments

***Phenylephrine (PE).*** To induce hypertrophy in vitro, primary cardiomyocytes from rat hearts were treated with PE (100 μM) for 4 days and followed by incubation in PE-free medium for 1 day as previous methods 18. The cardiomyocytes that treated with saline served as vehicle control.

***KMV.*** For EC treatment, cells were treated with KMV (20 μM) for 96 h. For *in vivo* treatment, mice received KMV (500 μg/kg/day, intraperitoneally) for 7 weeks. The dosages of KMV treatments were selected based on previous studies 22, 23.

***Human serum*.** ECs were treated with serum from human patients for 96 h.

***Meox2 knockdown.*** To knockdown Meox2 expression, primary ECs were transfected with siRNA targeting human Meox2 mRNA. ECs that transfected with scrambled RNA served as controls. The siRNA sequences are shown in **Table S6**.

### Paracrine effect of hypertrophic cardiomyocytes on EC angiogenesis

***Collecting conditioned medium (CM) from cardiomyocyte cultures.*** (1) For cardiomyocyte hypertrophy induced *in vivo*, the cardiomyocytes were isolated from mouse hearts after TAC for 7 weeks, cultured for 24 h. The culture medium was then collected as CM. (2) For cardiomyocyte hypertrophy induced* in vitro*, primary rat cardiomyocytes were treated with PE for 4 days, followed by incubation in PE-free medium for another 24 h. The culture medium was then collected as CM.

***EC treatment with cardiomyocyte CM.*** Primary ECs were treated with cardiomyocyte CM for 96 h followed by analysis of EC angiogenic behaviors.

### Nontargeted metabolomics

To compare metabolite secretion between control and hypertrophic cardiomyocytes, the culture medium of cardiomyocytes was collected for nontargeted metabolomics (Panomix Biotech, China) by using LC-MS analysis. The detailed methods were described in previous studies 46. All data were analyzed using BioDeep cloud platform (Panomix Biotech, China).

### EC angiogenesis in vitro

***EC proliferation.*** It was analyzed by EdU incorporation assay according to our previous methods 47. Briefly, primary ECs were incubated with EdU for 2 h by using the assay kit, and EC proliferation was expressed as the percentage of Edcells over total cells. Additionally, an MTT assay was performed to evaluate cell viability according to our previous methods 48.

***EC migration.*** It was analyzed by wound healing assay according to our previous methods 49. Briefly, primary ECs were grown in a six-well plate. After the indicated treatments, a linear scratch was made across the plate to create a streak wound using a 200 μl pipette. Progression of migration was observed and photographed at 0 and 12 h after scratch and expressed as the relative closure areas using Image J software (National Institutes of Health, Bethesda, MD).

***Tube formation***. It was analyzed according to our previous methods 44. Briefly, primary ECs (1.5 × cells/well) were seeded on growth factor-reduced matrigel-coated 96-well plates. Cells were photographed at 4 h after grown on Matrigel using a microscope. Tube formation was expressed as total branch length/field and master junctions/field using NIH ImageJ software.

### MTT assay

MTT assays were conducted to assess the viability of ECs following DHMA treatment, as described in our previous study 50. Briefly, ECs were incubated with MTT (0.5 mg/mL) for 4 h, followed by the addition of DMSO for 15 min to dissolve the formazan crystals. The optical density (OD) values were measured at 570 nm using a Synergy HT plate reader (BioTek, USA).

### KMV examination by ELISA Kit

The concentration of KMV was examined using an ELISA Kit according to manufacturer's instructions. A four-parameter logistic model was used to generate the standard curve, from which the concentration values were determined corresponding to each OD value.

### SA-β-gal Staining

It was examined using the SA-β-gal staining kit. Briefly, ECs were fixed with 4% PFA for 15 minutes followed by incubated with mixed staining solution overnight at 37 °C. The staining was photographed using a microscope.

### Quantitative Real-Time PCR (q-PCR)

The real expression of aimed genes was examined by q-PCR according to our previous methods 51. In brief, total mRNA was isolated for cDNA synthesis and q-PCR was performed using SYBR Green Master. The primers were shown in **Table S7**.

### Immunoblotting

Whole-cell lysates and nuclear protein extracts were prepared from mouse hearts or primary ECs for immunoblotting as previous methods 51. Immunoblotting for β-Actin and histone served as loading controls for whole cell lysates and nuclear protein extracts, respectively. The developed bands were expressed as relative integrated intensities (normalized to controls). Antibodies used in this experiment are listed in **Table S8**.

### Immunofluorescence staining

Heart tissues at papillary muscle level were prepared for frozen sectioning. Kidney and lung tissues were also collected for frozen sectioning. Retina tissues were collected for whole-mount staining according to previous methods 52. Primary ECs and primary cardiomyocytes were harvested. After fixation with 4% PFA, the samples were immunostained as our previous methods 42, 51. Briefly, the samples were incubation with the indicated primary antibody (1:100) overnight at 4 °C, followed by incubated with Cy3- or Alexa Fluor 488-conjugated secondary antibodies. DAPI was used to counterstain the nuclei. The staining was observed by using a fluorescence microscope and quantified using Cellsens Dimension 1.15 software (Olympus, Tokyo, Japan).

### Microvascular blood flow phenomenon in hearts

Cardiac microvascular blood flow was evaluated using FITC-labeled *Lycopersicon esculentum* (Tomato) lectin (Vector Laboratories, Newark, CA) according to the method described previously 53, 54. Briefly, FITC-labeled Tomato lectin (25 μL) was intravenously injected into mice following TAC or TAC+KMV treatment for 7 weeks. After allowing 15 min for circulation, transverse-sectioning at papillary muscle level was prepared and analyzed by fluorescence microscopy (Olympus, Tokyo, Japan). The blood flow area was indicated by staining of FITC-labeled Tomato lectin.

### RNAsequencing (RNA-Seq)

Following KMV treatment for 96 h, primary ECs were collected for RNA-Seq analysis by Panomix Biotech (China). The detailed methods were described previously 55. Differential expressed genes (DEGs) between groups were identified using DEGSeq (v 3.14) with |log2 fold change| > 1 and p < 0.05. GO enrichment was analyzed with TopGO (v 2.14.0).

### Mendelian randomization (MR) study

The data were obtained from the GWAS Catalog (https://www.ebi.ac.uk/gwas/). The causal relationship between KMV and Vascular/heart problems diagnosed by high blood pressure was explored using two-sample MR. MR analyses were conducted using the R package "TwoSampleMR" 56. SNPs strongly associated with exposure factors were selected as instrumental variables based on the following criteria:* P*-value less than 5 × , R-squared value less than 0.1, and length of 10,000. This approach was adopted to ensure the absence of weak instrument bias.

### Chromatin immunoprecipitation (ChIP)-PCR

Following KMV treatment for 96 h, primary ECs were harvested for anti-Meox2 chromatin immunoprecipitation using a ChIP assay kit. Detailed ChIP methods were described in the Supplementary Methods. PCR was performed using Meox2 ChIP immunocomplex. The PCR products were separated on 2% agarose gel. The amplification contained putative Meox2 binding sites at the promoter region (-115/-106) of *p16*, the promoter region (-709/-703) of *p21* gene, and the promoter region (-462/-453) of *Vegf-a* gene. The primers used for PCR are listed in **Table S9**.

### Statistical analysis

Data are presented as mean ± standard deviation (SD). Groups were compared using Student's two-tailed paired *t*-test, one-way ANOVA or two-way ANOVA followed by Tukey's test as a post-hoc analysis. Survival analysis was performed using the log-rank test. A *P*-value < 0.05 was considered statistically significant.

## Supplementary Material

Supplementary figures and tables.

## Funding

This study was supported by the National Natural Science Foundation of China (82471624, 82470411 82170295, 82170851, and 82000296), by a project funded the Key Laboratory of Targeted Intervention of Cardiovascular Disease, Collaborative Innovation Center for Cardiovascular Disease Translational Medicine, Nanjing Medical University, Jiangsu Provincial Key Research and Development Program (BE2023818), and by the Scientific Research Project of Jiangsu Commission of Health (LKZ2023001).

## Abbreviations

Ang-1: angiopoietin-1
BCAA: branched-chain amino acid
BCKA: branched-chain keto acid
ChIP: chromatin immunoprecipitation
CM: conditioned medium
DEG: differentially expressed gene
EC: endothelial cell
ELISA: enzyme-linked immunosorbent assay
HF: heart failure
KMV: 3-methyl-2-oxovaleric acid
Meox2: mesenchyme homeobox 2
PE: phenylephrine
PO: pressure overload
RNA-seq: RNA sequencing
TF: transcription factor
VEGF: vascular endothelial growth factor
