# Supplementary material for: KMV-mediated cardiomyocyte-to-endothelial cell signaling drives capillary rarefaction to promote heart failure following pressure overload
Source: Theranostics. 2025 Mar 31;15(11):4970–88. doi: 10.7150/thno.104899 (PMC12036891; doi:10.7150/thno.104899)
Supplement: Supplementary file 1 — Supplementary figures and tables. [file thnov15p4970s1.pdf]

Supplemental materials

Legends for supplemental figures

Figure S1. TAC decreased cardiac diastolic dysfunction in mice.

Echocardiography was conducted to examine cardiac E/e’ ratio after sham or TAC for 7 weeks in mice. Data are mean ± SD, ** P < 0.01 by Student’s two-tailed unpaired t-test. n = 6/group.

Figure S2. Conditioned medium (CM) from hypertrophic cardiomyocyte cultures impaired angiogenesis *in vitro*.

A. **Experimental setting.** Following hypertrophy was induced by PE, CM was collected from cardiomyocyte culture for EC treatment.

B. **Cardiomyocyte hypertrophy.** Following hypertrophy induction by PE, cardiomyocyte size was examined by immunostaining for α-Actinin. DAPI was used to satin nuclei. Scale bar = 10 μm.

C-E. **Effect of cardiomyocyte CM on endothelial angiogenesis.** Following treatment with cardiomyocyte CM for 96 h, ECs were subjected to proliferation assay by EdU incorporation (**C**, Scale bar = 100 μm), migration assay by wound closure (**D**, Scale bar =

200 μm), and tube formation on Matrigel growth (**E**, Scale bar = 200 μm).

F. Effect of cardiomyocyte CM on angiogenic factor expression in ECs. VEGF and Ang-1 expression was determined by immunoblotting.

Data are mean ± SD, ** $P < 0.01$ by Student's two-tailed unpaired *t*-test (**B-C**, **E-F**) and two-way ANOVA followed by Tukey's test (**D**). n = 6/group.

Figure S3. KMV concentration in mouse serum.

Mouse serum was collected for KMV measurement after sham, KMV, or TAC treatment for 7 weeks. Data are mean ± SD, **$P < 0.01$ by one-way ANOVA followed by Tukey's test. n = 6/group.

Figure S4. Mendelian randomization (MR) analysis uncovered the opposite correlation between KMV and vascular diseases.

A. Forest plot. Forest plot of the relationship between the level of KMV (3-Methyl-2-oxovaleric acid) and vascular/heart problems diagnosed by high blood pressure by MR analysis.

B. Scatter plots. Scatter plot for MR analysis of the causal effect of the level of KMV (3-Methyl-2-oxovaleric acid) on vascular/heart problems diagnosed by High blood pressure by MR analysis.

Figure S5. The serum from heart failure (HF) patients impaired angiogenesis *in vitro*.

A. Experimental diagram. After treatment with serum of HF patients and non-HF controls for 96 h, ECs were subjected to the following analyses.

B. EC proliferation. EdU incorporation was performed to examine EC proliferation. Scale bar = 100 μm. n = 6/group.

C. Tube formation. The tube formation of ECs was examined by growing on Matrigel. Scale bar = 200 μm. n = 6/group.

D. EC migration. EC migratory ability was examined by wound healing assay and expressed as the percentage of wound closure. Scale bar = 200 μm. n = 6/group.

E. Expression of VEGF and Ang-1. The indicated protein expression was examined by immunoblotting. n = 3/group.

Data are mean ± SD, ** $P < 0.01$ by Student's two-tailed unpaired *t*-test (**B-C, E**) and two-way ANOVA followed by Tukey's test (**D**).

Figure S6. Effects of KMV on VE-cadherin and p-VE-cadherin expression in ECs.

A. Experiment diagram. Primary ECs were treated with KMV, conditioned medium, or HF serum for 96 h, followed by analysis of related proteins expression.

B-D. **p-VE-cadherin and VE-cadherin expression.** After different treatments for 96 h, the expression of p-VE-cadherin and VE-cadherin was examined using immunoblotting. Data are mean ± SD, **$P < 0.01$ by Student's two-tailed unpaired *t*-test. n = 6/group.

Figure S7. Effects of DHMA on EC angiogenesis.

A-C. EC proliferation. The dose-effect (**A**) and time-effect (**B**) of DHMA on EC viability was examined by MTT assay. The EDU assay was used to evaluate EC proliferation (**C**, scale bar = 100 μm). n = 6/group.

D. EC migration. EC migratory ability was examined by a wound healing assay and expressed as the percentage of wound closure. Scale bar = 200 μm. n = 6/group.

E. Tube formation. The tube formation of ECs was examined by growing them on Matrigel. Scale bar = 200 μm. n = 6/group.

F. Angiogenic factor expression. The indicated protein expression was examined by immunoblotting. n = 3/group.

Data are presented as mean ± SD, analyzed by one-way ANOVA followed by Tukey's test (**A-B**), student's two-tailed unpaired *t*-test (**C, E-F**), or two-way ANOVA followed by Tukey's test (**D**).

Figure S8. Effects of KMV on VEGFR2 expression in TAC hearts.

After 7 weeks of treatment with sham, TAC, or TAC + KMV, heart tissues were collected for immunoblotting analysis of VEGFR2 (**A**). Additionally, heart tissues at the papillary muscle level were frozen-sectioned for co-staining of VEGFR2 and CD31 (**B**, scale bar = 10 μm). Data are mean ± SD, $^{**}P < 0.01$ by one-way ANOVA followed by Tukey's test. n = 6/group.

Figure S9. Effect of KMV on microvascular blood flew phenomenon in TAC heart.

Cardiac microvascular blood flow was assessed by FITC-labeled *Lycopersicon esculentum* (Tomato) lectin. The blood flow area was determined by the staining pattern of FITC-labeled Tomato lectin. Scale bar = 500 μm (upper panels) and 50 μm (down panels). Data are mean ± SD, $*P < 0.05$ and $**P < 0.01$ by one-way ANOVA followed by Tukey's test. n = 4/group.

Figure S10. The E/e' ratio in heart of mice.

Echocardiography was performed to assess the cardiac E/e' ratio in mice after 7 weeks of sham, TAC, or TAC + KMV treatment. Data are mean ± SD, $** P < 0.01$ by one-way ANOVA followed by Tukey's test. n = 6/group.

Figure S11. Effects of KMV on angiogenesis in hearts, retinas, kidneys, and lungs in mice without TAC.

Mice without TAC (sham) were treated with KMV or saline for 7 weeks. Following treatment, the heart, retina, kidney, and lung were collected for immunostaining against CD31, with DAPI used to counterstain nuclei. Scale bars: (**A**) Heart: 100 μm (upper panels) and 50 μm (lower panels); (**B**) Retina: 200 μm (upper panels) and 50 μm (lower panels); (**C**) Kidney: 50 μm (upper panels) and 20 μm (lower panels); (**D**) Lung: 50 μm (upper panels) and 20 μm (lower panels). Data are mean ± SD, $**P < 0.01$ by Student's two-tailed unpaired *t*-test. n = 6/group.

Figure S12. The effect of KMV on the expression of angiogenic factors in retinas, kidneys, and lungs in mice without TAC.

Mice without TAC (sham) were treated with KMV or saline for 7 weeks. Following treatment, tissues from the heart, retina, kidney, and lung were collected for immunoblotting to detect the indicated proteins. Data are mean ± SD, $^{**}P < 0.01$ by Student's two-tailed unpaired *t*-test. n = 6/group.

Figure S13. The effect of KMV on cardiomyocyte size and cardiac fibrosis in mice without TAC.

Mice without TAC (sham) were treated with KMV or saline for 7 weeks. Following treatment, heart tissues were collected for the following analyses:

A. WGA staining. Cardiac frozen sections were prepared for WGA staining (green) to assess cardiomyocyte size. Nuclei were counterstained with DAPI (blue). Scale bars: 50 μm (upper panels) and 20 μm (lower panels).

B. The expression of fetal genes. ANP expression was analyzed by immunoblotting.

C-D. Cardiac fibrosis. Collagen III expression was evaluated in hearts using immunofluorescence staining (**C,** scale bar = 20 μm.) and immunoblotting (**D**).

Data are mean ± SD, $^{**}P < 0.01$ by Student's two-tailed unpaired *t*-test. n = 6/group.

Figure S14. TAC increased Meox2 expression in ECs of hearts.

A. **Experimental diagram.** After TAC or sham surgery for 7 weeks, hearts were harvested for the following analyses.

B. **Immunoblotting.** Protein expression of Meox2 was examined in cardiac protein extracts using immunoblotting.

C. **Immunostaining.** Cardiac frozen sections were prepared for immunostaining against Meox2 (green) and CD31 (red). DAPI was used to counterstain nuclei. Arrows indicate the Meox2 expression in ECs of mouse hearts. Scale bar = 5 μm.

Data are mean ± SD, ** $P < 0.01$ by Student's two-tailed unpaired *t*-test. n = 6/group.

Figure S15. Conditioned medium (CM) from hypertrophic cardiomyocyte cultures induced endothelial senescence.

A. **Experimental diagram.** Following the induction of hypertrophy by PE, CM was collected from cardiomyocyte cultures and used to treat ECs for 96 h.

B. **SA-β-gal Staining.** SA-β-gal staining was performed to indicate EC senescence and expressed as the percentage of β-gal^{+} ECs over total ECs. Scale bar = 50 μm, n = 3/group.

C. **Immunoblotting.** Expression of p16, p21, and p53 was examined by immunoblotting. n = 6/group

Data are mean ± SD, ** $P < 0.01$ by Student's two-tailed unpaired *t*-test.

Figure S16. Effects of other transcription factors (TFs) in KMV-induced EC senescence.

A. **Effect of KMV on TF expression.** Following KMV treatment for 96 h, ECs were collected for immunoblotting against the indicated TFs. n = 3/group

B. **TBX1 knockdown.** TBX1 knockdown was confirmed by immunoblotting. n = 6/group.

C-D. Effects of TBX1 knockdown on KMV-induced EC senescence. KMV was introduced to control and TBX1 knockdown ($Tbx1^{KD}$) primary ECs for 96 h. After then, ECs were subjected to SA-β-gal staining (**C**, scale bar = 50 μm) and immunoblotting against p16 and p21 (**D**). n = 3/group

Data are mean ± SD, $^{**}P < 0.01$ by Student's two-tailed unpaired *t*-test (**A, C-D**) and by one-way ANOVA followed by Tukey's test (**B**).

Figure S17. A brief overview of the isolation and plating procedure for cardiomyocytes from adult mice heart.

A. **Aortic Cannulation and Heart Perfusion.** The heart was removed from euthanized adult mice, the aorta was cannulated, and the heart was perfused to digest the myocardial tissue.

B. **Mincing the Heart.** After digestion, the heart was minced into small pieces using scissors.

C. **Dissociating Cardiomyocytes.** The minced heart tissue was gently triturated (by pipetting or swirling) to release individual cardiomyocytes into the suspension.

D. **Pre-Plating the Isolated Adult Cardiomyocytes.** The isolated adult cardiomyocytes were preplated for 1 h to allow the attachment of viable cardiomyocytes.

E. **Removing Dead Cells.** After 1 h of incubation, the culture medium was changed to

remove non-viable cells. The image shows the attached viable cardiomyocytes.

Figure S18. A brief overview of the isolation and plating procedure for primary cardiomyocytes from neonatal rat hearts.

A. **Heart Collection.** Hearts were collected from euthanized neonatal rats.

B. **Mincing the Heart.** Hearts were minced into small pieces using scissors.

C. **Digestion.** The minced heart tissues were digested with enzymes to release individual cells.

D. **Cell Culture.** The released cell suspension was centrifuged to pellet the cells. The pelleted cells were resuspended and preplated for 1 h to allow the attachment of non-cardiomyocytes.

E. **Non-Cardiomyocytes.** After 1 h of preplating, the attached cells were considered non-cardiomyocytes.

F. **Cardiomyocytes.** After 1 h of preplating, the unattached cells, which were considered cardiomyocytes, were transferred to another culture dish. The image shows cardiomyocytes after 24 h of culture.

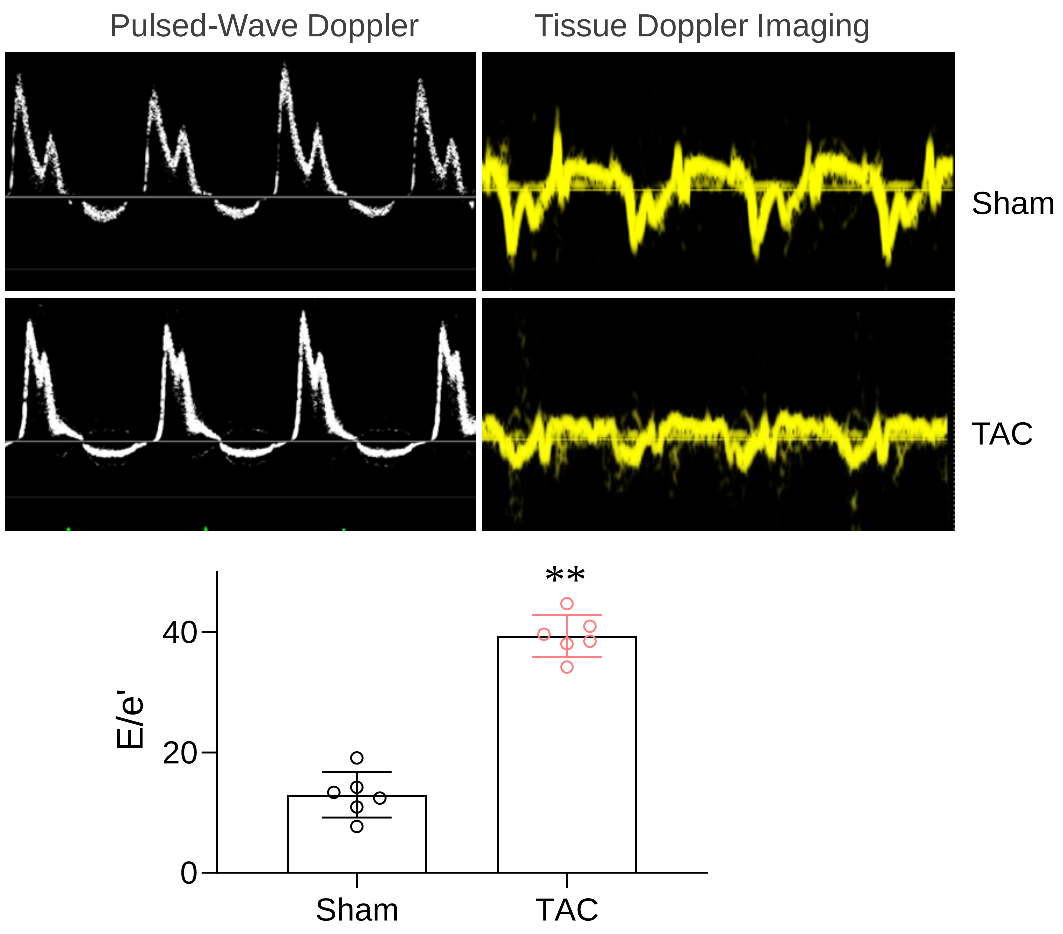
Pulsed-Wave Doppler
Tissue Doppler Imaging
Sham
TAC
**
40
20
0
E/e'
Sham
TAC

Figure S1

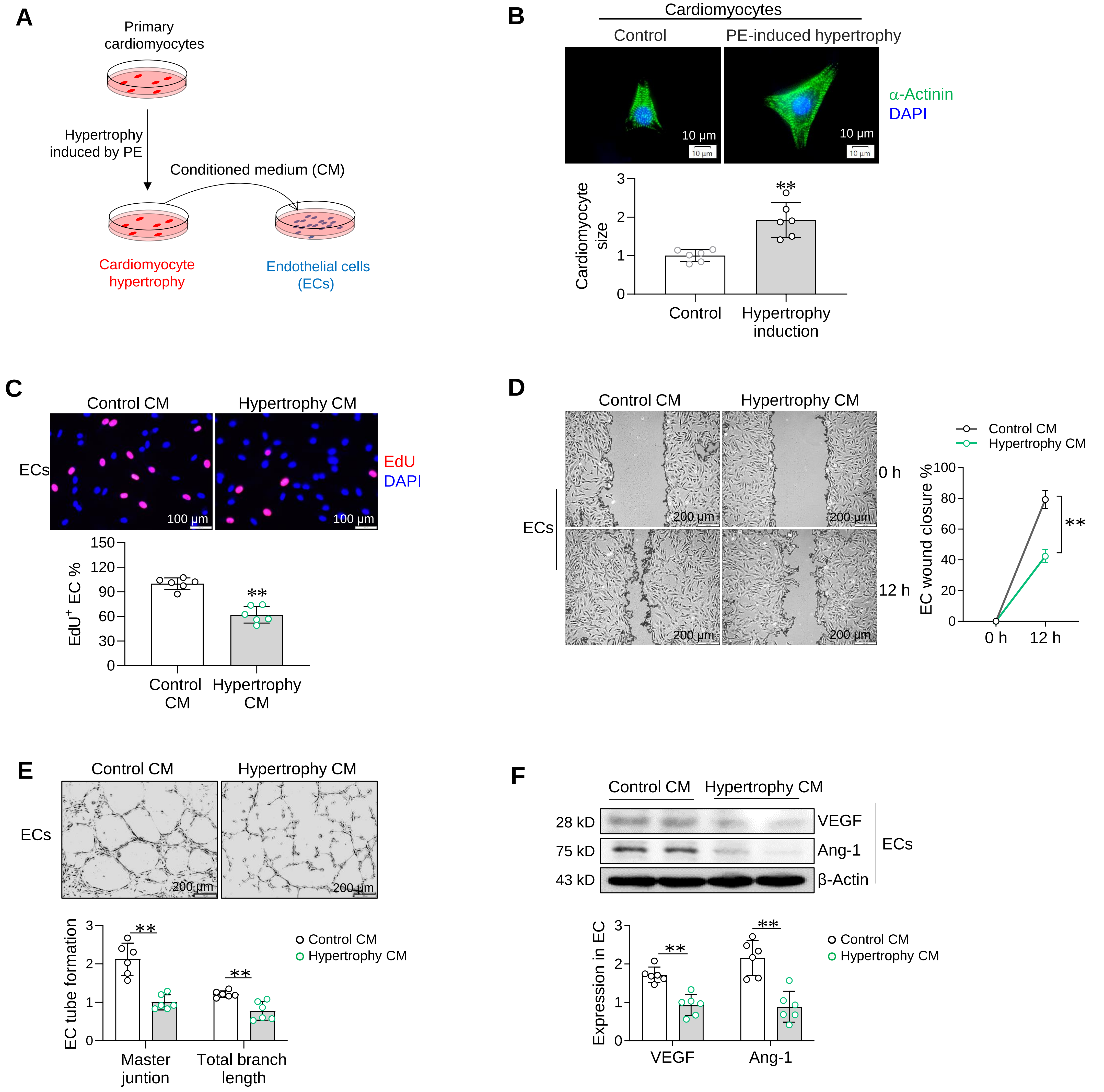

A
Primary cardiomyocytes
Hypertrophy induced by PE
Conditioned medium (CM)
Cardiomyocyte hypertrophy
Endothelial cells (ECs)
B
Cardiomyocytes
Control
PE-induced hypertrophy
α-Actinin
DAPI
10 μm
Cardiomyocyte size
Control
Hypertrophy induction
**
C
Control CM
Hypertrophy CM
ECs
EdU
DAPI
100 μm
EdU+ EC %
Control CM
Hypertrophy CM
**
D
Control CM
Hypertrophy CM
ECs
0 h
12 h
200 μm
EC wound closure %
**
E
Control CM
Hypertrophy CM
ECs
200 μm
EC tube formation
Master juntion
Total branch length
**
F
Control CM
Hypertrophy CM
28 kD
75 kD
43 kD
VEGF
Ang-1
β-Actin
ECs
Expression in EC
**

Figure S2

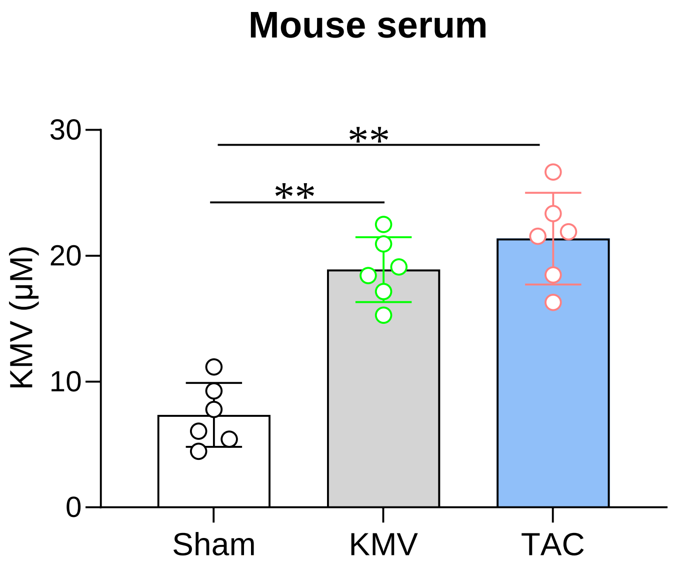

Figure S3

A

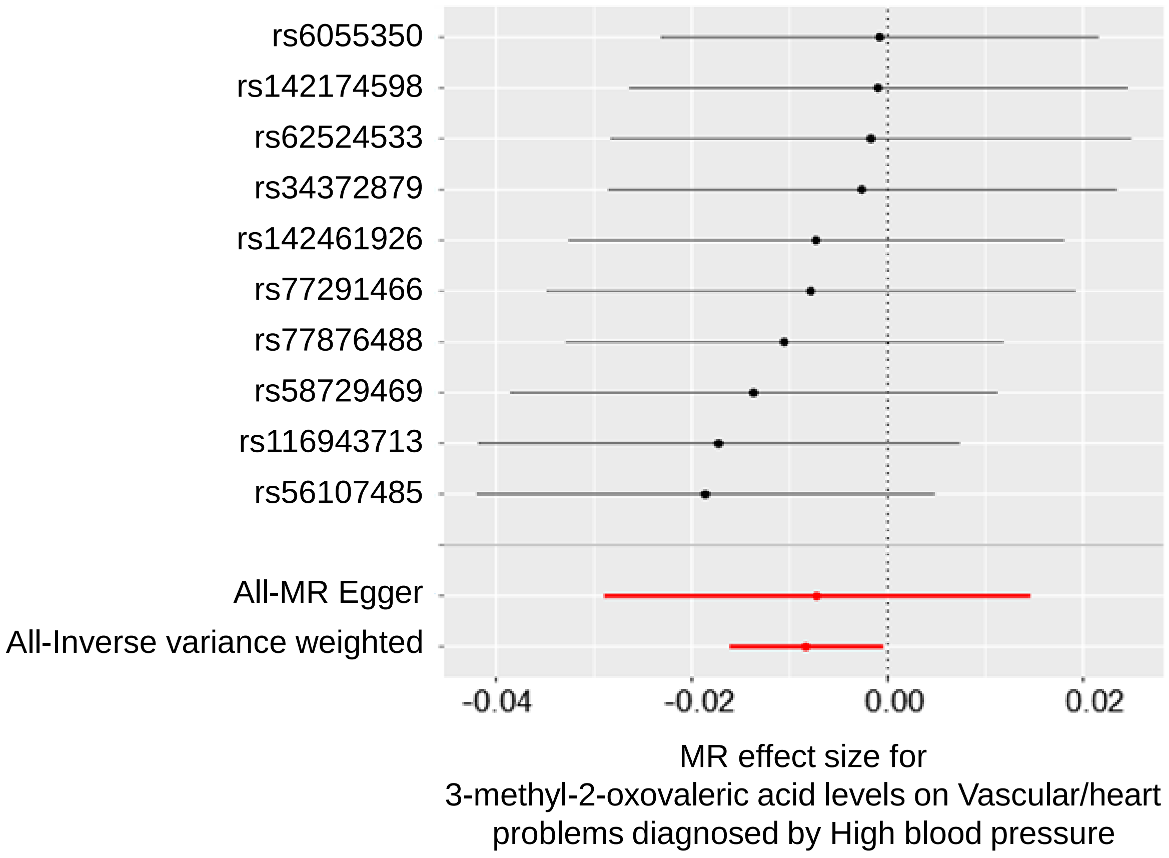
rs6055350
rs142174598
rs62524533
rs34372879
rs142461926
rs77291466
rs77876488
rs58729469
rs116943713
rs56107485
All-MR Egger
All-Inverse variance weighted
-0.04
-0.02
0.00
0.02
MR effect size for
3-methyl-2-oxovaleric acid levels on Vascular/heart
problems diagnosed by High blood pressure

B

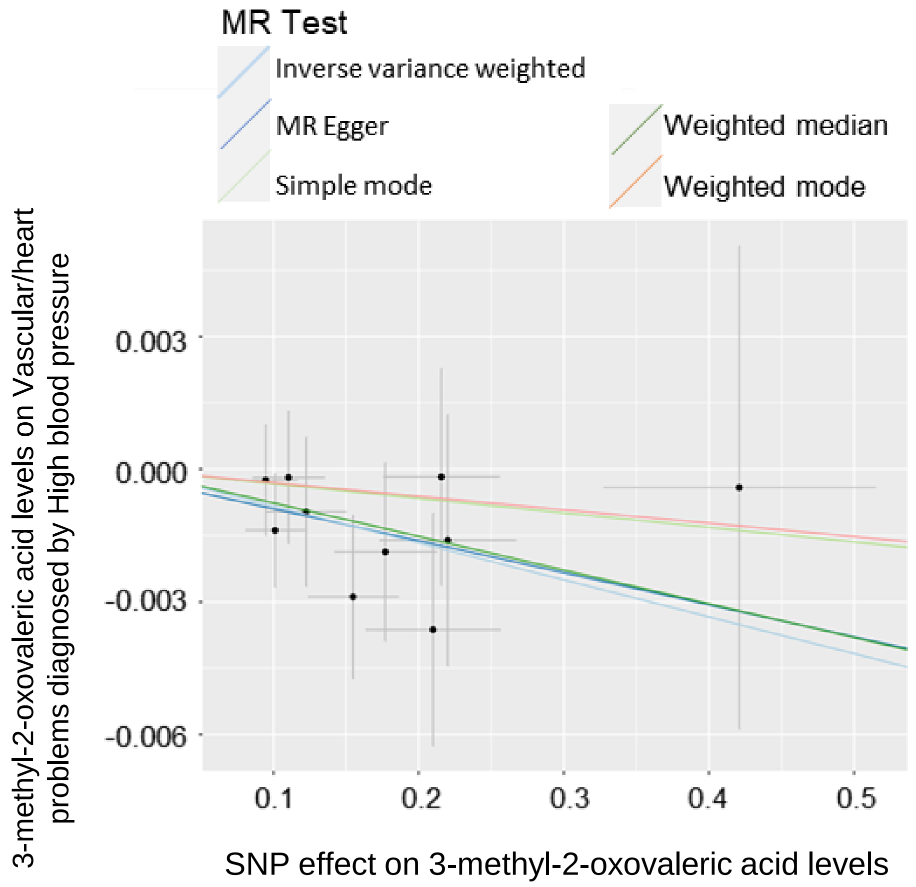
MR Test
Inverse variance weighted
MR Egger
Simple mode
Weighted median
Weighted mode
0.003
0.000
-0.003
-0.006
0.1
0.2
0.3
0.4
0.5
3-methyl-2-oxovaleric acid levels on Vascular/heart
problems diagnosed by High blood pressure
SNP effect on 3-methyl-2-oxovaleric acid levels

Figure S4

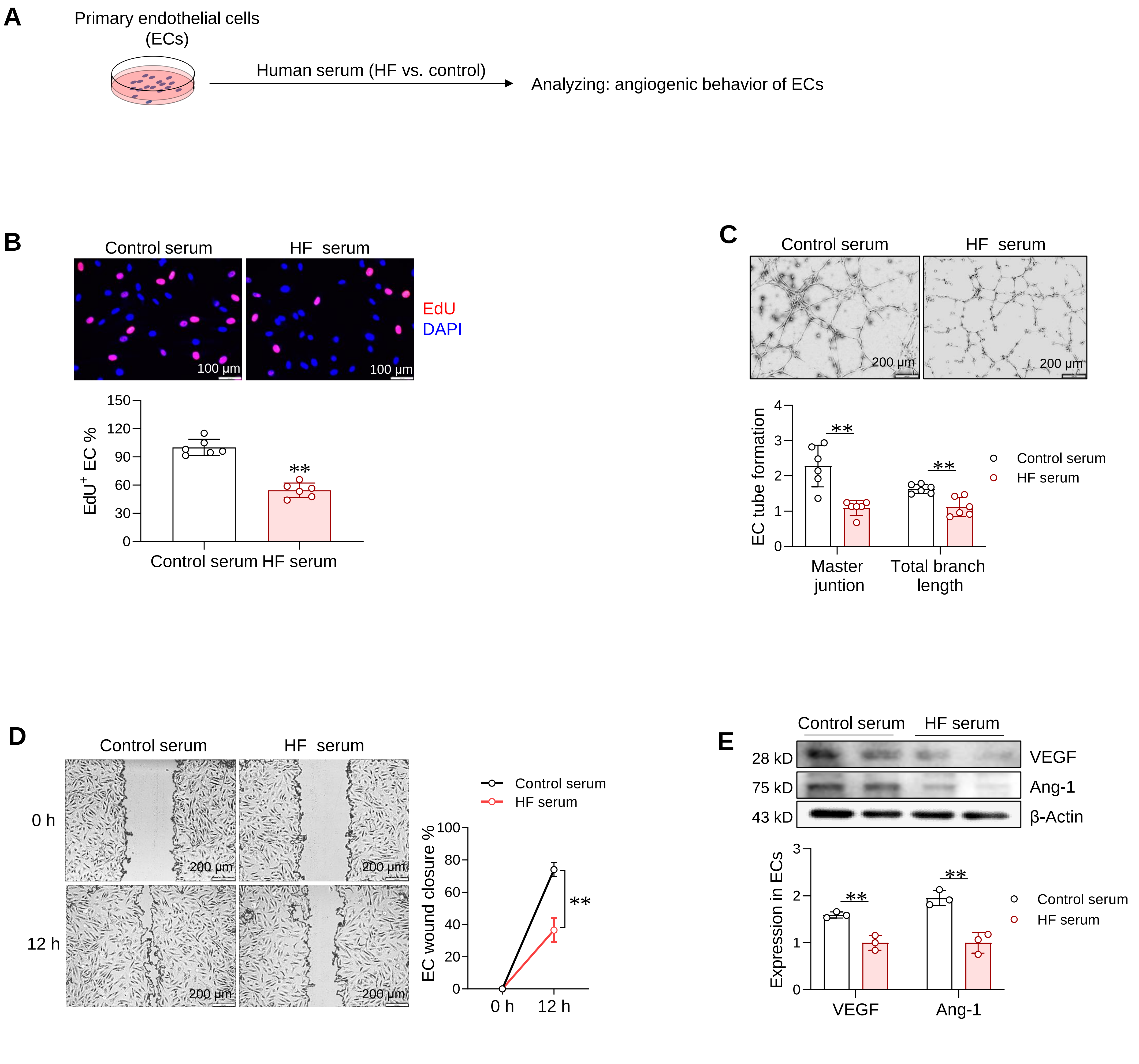
A
Primary endothelial cells
(ECs)
Human serum (HF vs. control)
Analyzing: angiogenic behavior of ECs
B
Control serum
HF serum
EdU
DAPI
100 µm
100 µm
EdU+ EC %
Control serum HF serum
C
Control serum
HF serum
200 µm
200 µm
EC tube formation
Master juntion
Total branch length
Control serum
HF serum
D
Control serum
HF serum
0 h
12 h
200 µm
EC wound closure %
0 h
12 h
E
Control serum
HF serum
28 kD
75 kD
43 kD
VEGF
Ang-1
β-Actin
Expression in ECs
VEGF
Ang-1

Figure S5

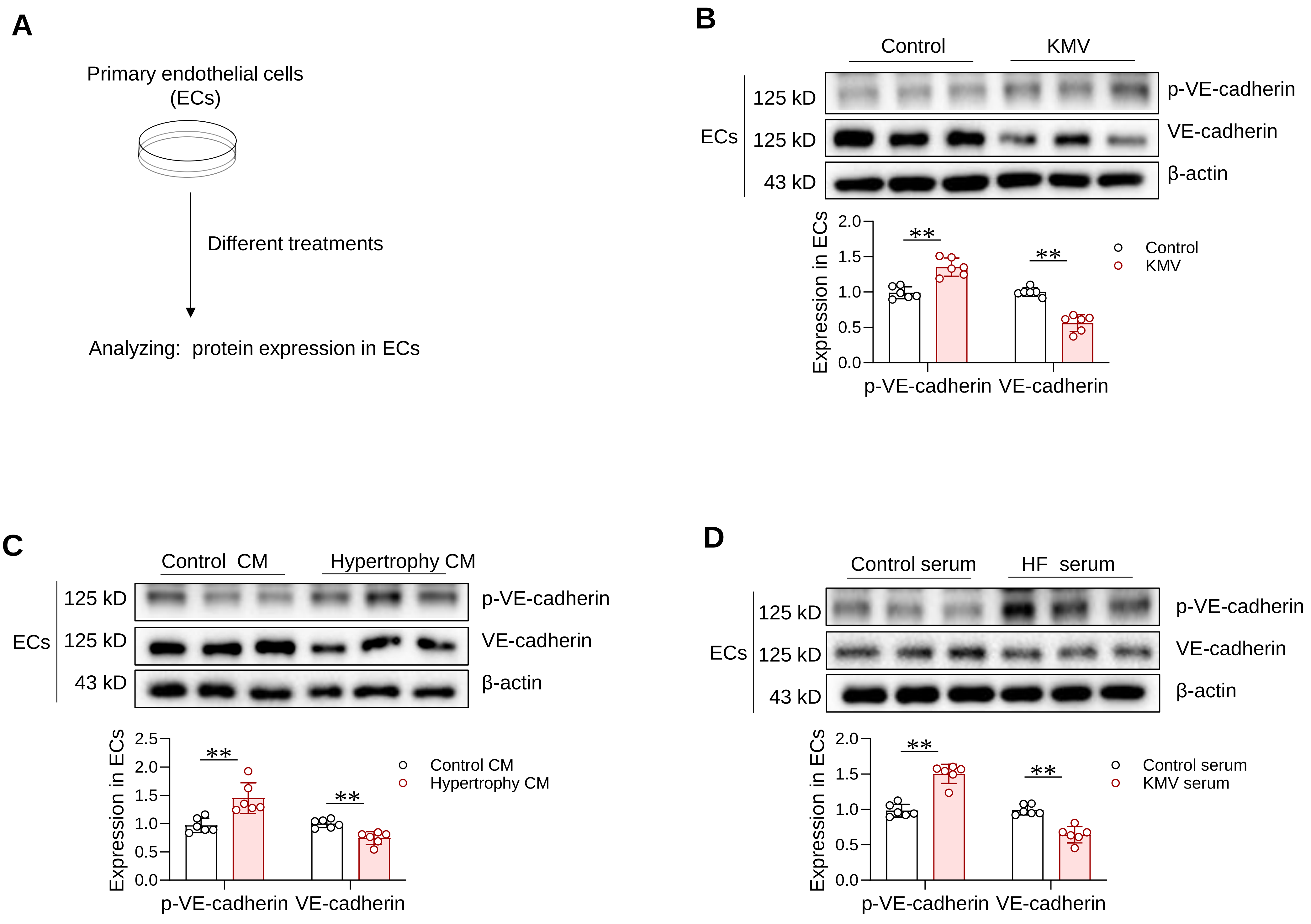
A
Primary endothelial cells
(ECs)
Different treatments
Analyzing: protein expression in ECs
B
Control
KMV
ECs
125 kD
125 kD
43 kD
p-VE-cadherin
VE-cadherin
β-actin
Expression in ECs
2.0
1.5
1.0
0.5
0.0
**
**
Control
KMV
p-VE-cadherin
VE-cadherin
C
Control CM
Hypertrophy CM
ECs
125 kD
125 kD
43 kD
p-VE-cadherin
VE-cadherin
β-actin
Expression in ECs
2.5
2.0
1.5
1.0
0.5
0.0
**
**
Control CM
Hypertrophy CM
p-VE-cadherin
VE-cadherin
D
Control serum
HF serum
ECs
125 kD
125 kD
43 kD
p-VE-cadherin
VE-cadherin
β-actin
Expression in ECs
2.0
1.5
1.0
0.5
0.0
**
**
Control serum
KMV serum
p-VE-cadherin
VE-cadherin

Figure S6

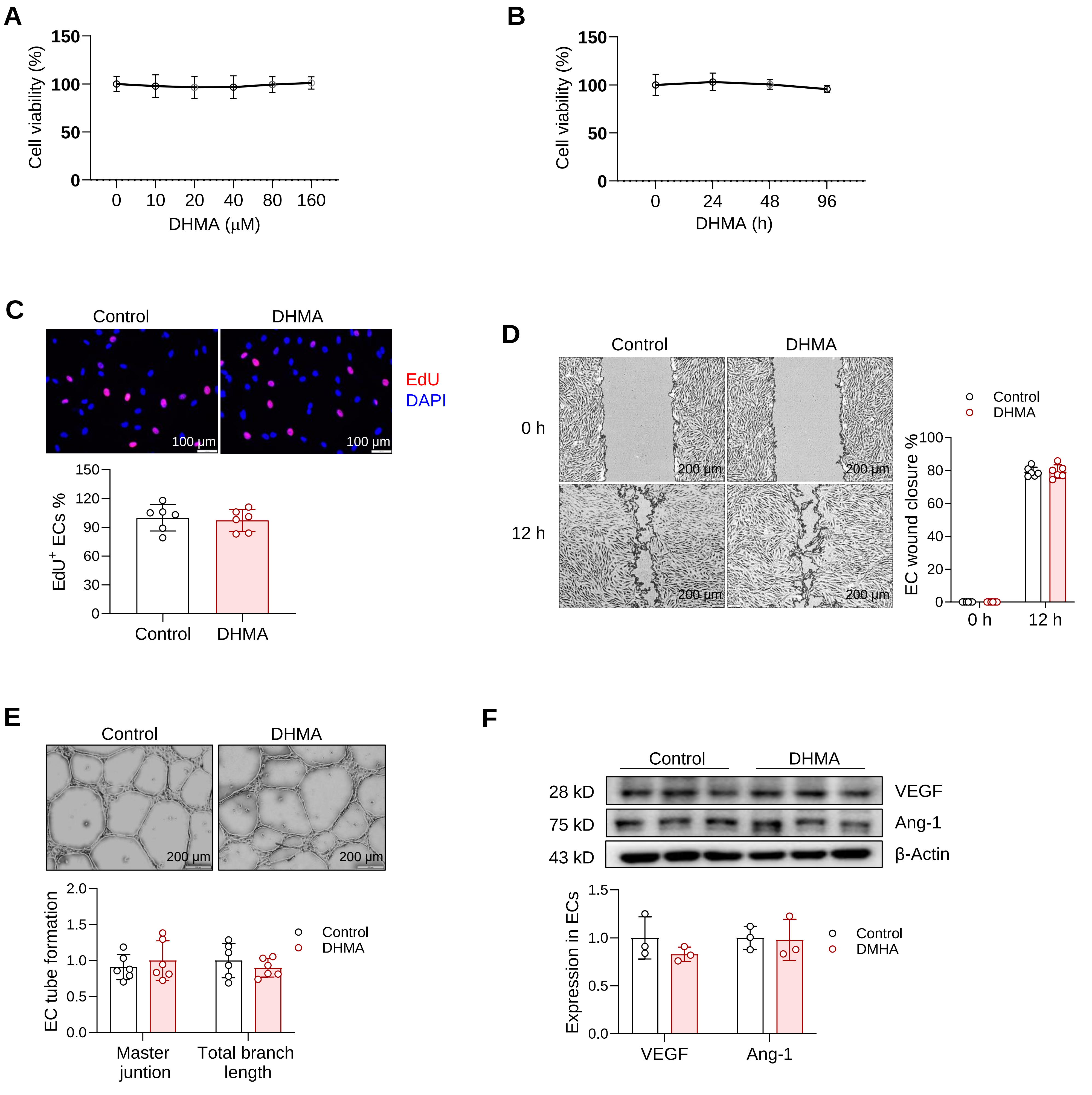
A
Cell viability (%)
150
100
50
0
0
10
20
40
80
160
DHMA (µM)
B
Cell viability (%)
150
100
50
0
0
24
48
96
DHMA (h)
C
Control
DHMA
EdU
DAPI
100 µm
100 µm
EdU+ ECs %
150
120
90
60
30
0
Control
DHMA
D
Control
DHMA
0 h
12 h
200 µm
Control
DHMA
EC wound closure %
100
80
60
40
20
0
0 h
12 h
E
Control
DHMA
200 µm
200 µm
EC tube formation
2.0
1.5
1.0
0.5
0.0
Control
DHMA
Master juntion
Total branch length
F
Control
DHMA
28 kD
75 kD
43 kD
VEGF
Ang-1
β-Actin
Expression in ECs
1.5
1.0
0.5
0.0
Control
DMHA
VEGF
Ang-1

Figure S7

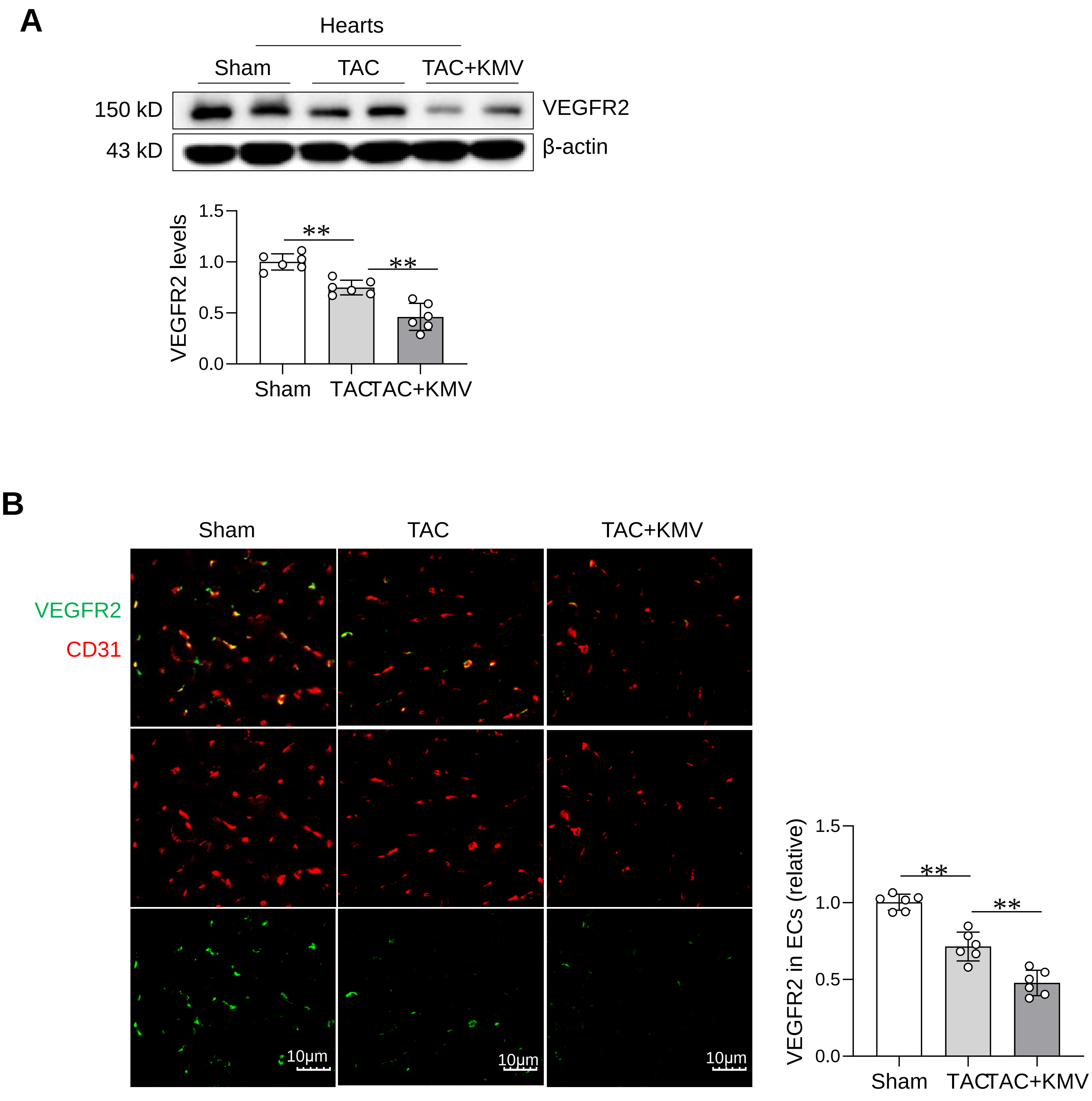
A
Hearts
Sham
TAC
TAC+KMV
150 kD
VEGFR2
43 kD
β-actin
VEGFR2 levels
1.5
1.0
0.5
0.0
**
**
Sham
TAC
TAC+KMV
B
Sham
TAC
TAC+KMV
VEGFR2
CD31
10μm
10μm
10μm
VEGFR2 in ECs (relative)
1.5
1.0
0.5
0.0
**
**
Sham
TAC
TAC+KMV

Figure S8

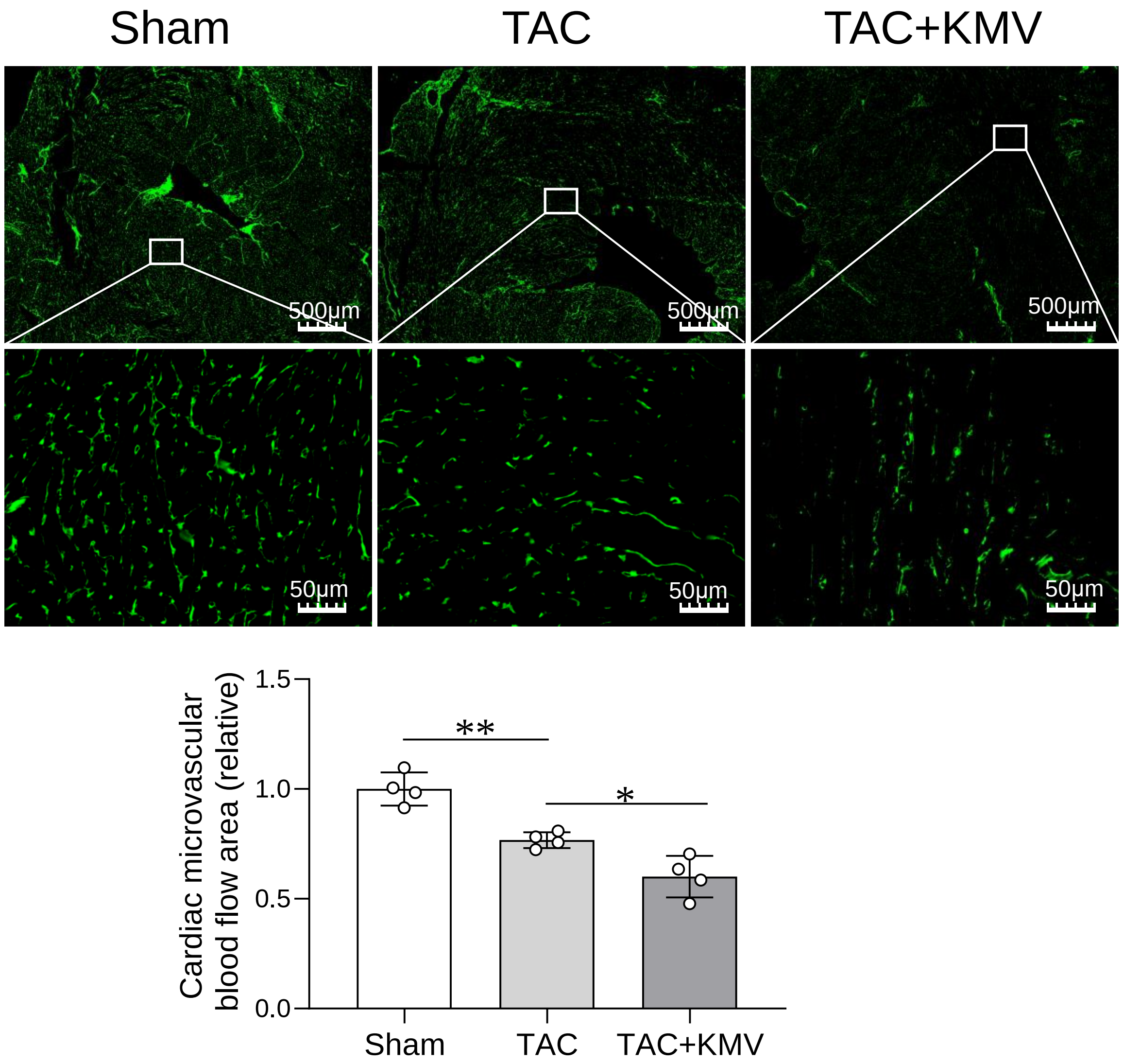

Sham
TAC
TAC+KMV
500μm
500μm
500μm
50μm
50μm
50μm
Cardiac microvascular blood flow area (relative)
1.5
1.0
0.5
0.0
**
*
Sham
TAC
TAC+KMV

Figure S9

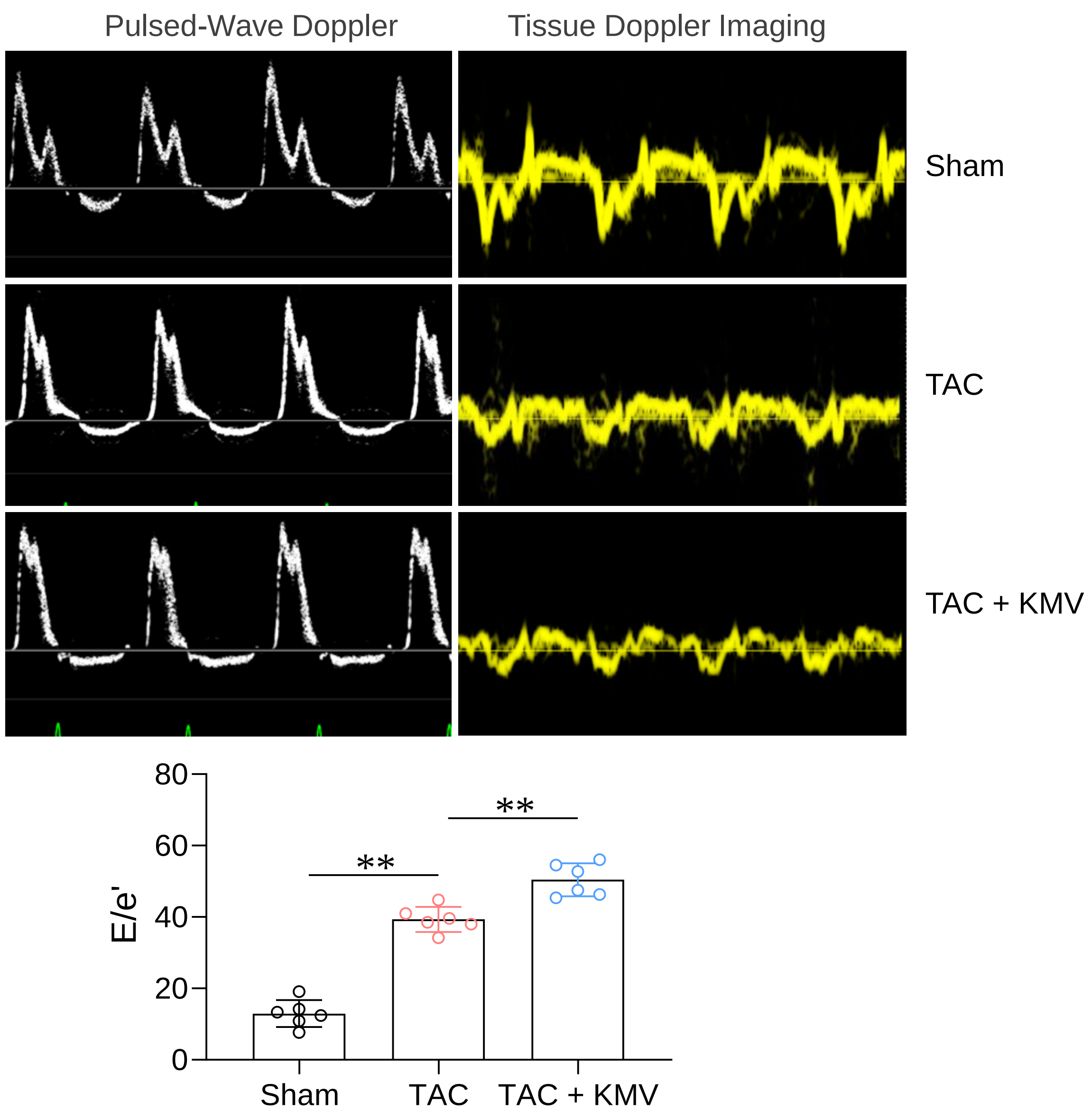
Pulsed-Wave Doppler
Tissue Doppler Imaging
Sham
TAC
TAC + KMV
80
60
40
20
0
E/e'
**
**
Sham
TAC
TAC + KMV

Figure S10

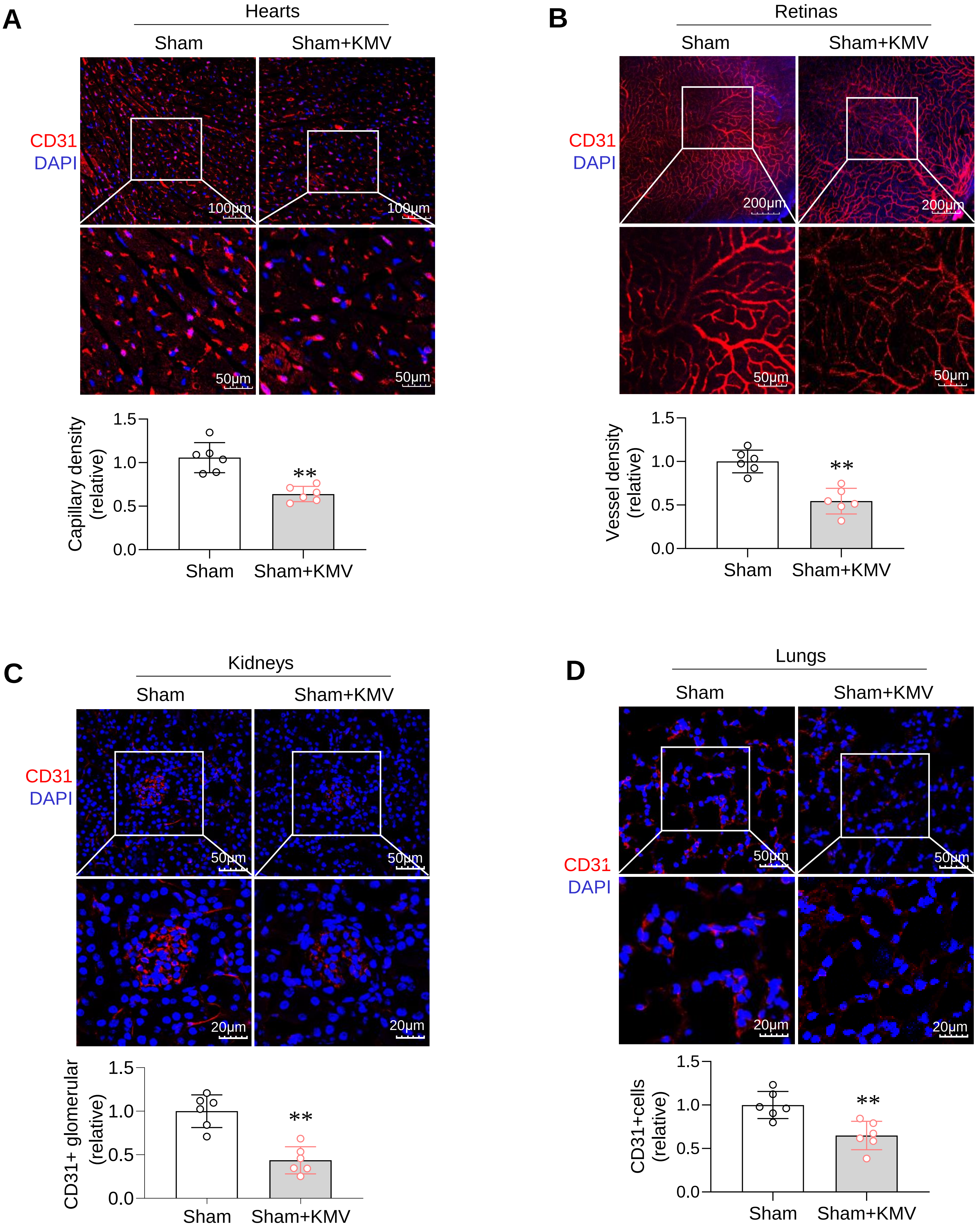
A
Hearts
Sham
Sham+KMV
CD31
DAPI
100μm
100μm
50μm
50μm
Capillary density (relative)
1.5
1.0
0.5
0.0
**
Sham
Sham+KMV
B
Retinas
Sham
Sham+KMV
CD31
DAPI
200μm
200μm
50μm
50μm
Vessel density (relative)
1.5
1.0
0.5
0.0
**
Sham
Sham+KMV
C
Kidneys
Sham
Sham+KMV
CD31
DAPI
50μm
50μm
20μm
20μm
CD31+ glomerular (relative)
1.5
1.0
0.5
0.0
**
Sham
Sham+KMV
D
Lungs
Sham
Sham+KMV
CD31
DAPI
50μm
50μm
20μm
20μm
CD31+cells (relative)
1.5
1.0
0.5
0.0
**
Sham
Sham+KMV

Figure S11

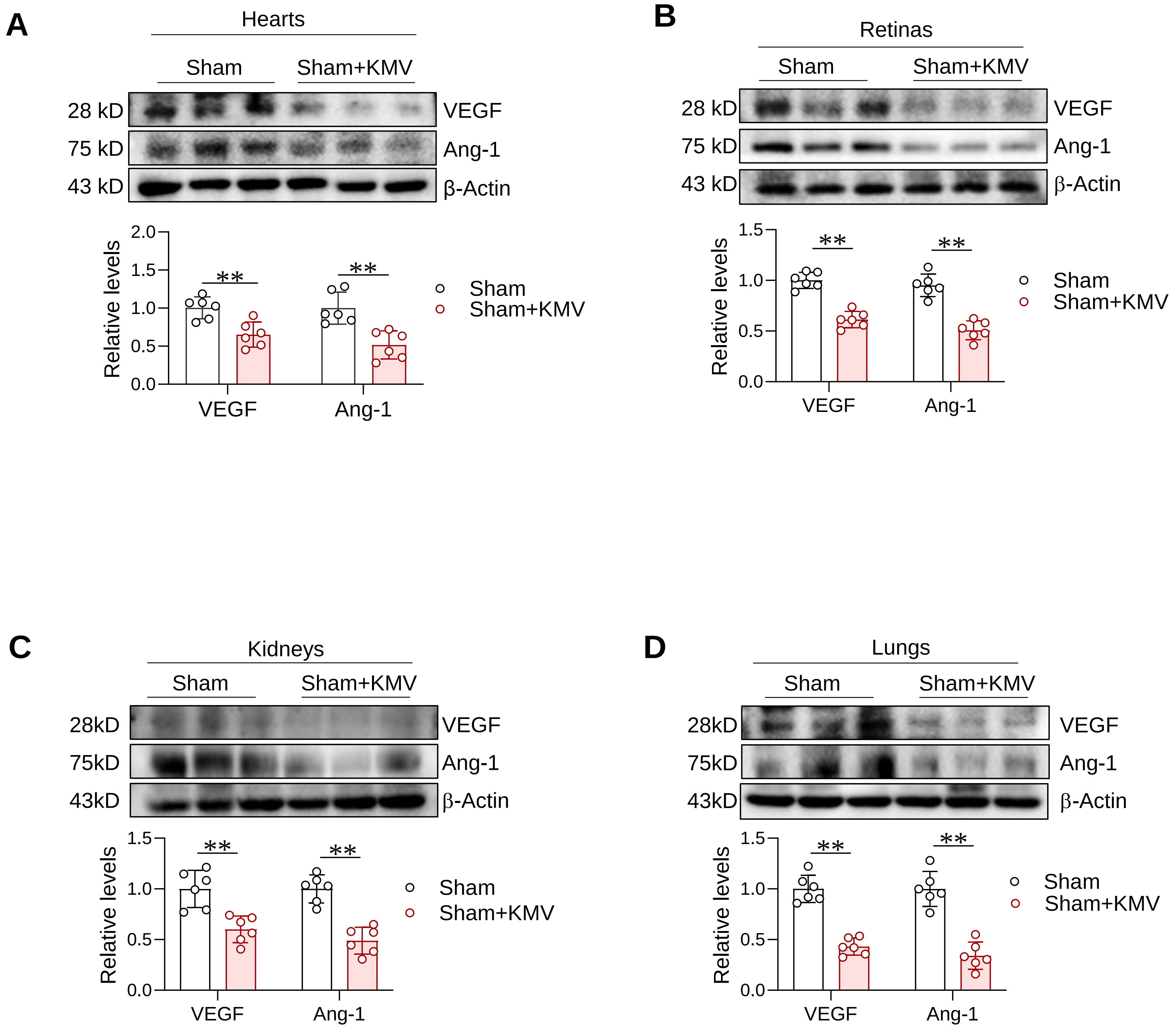
A
Hearts
Sham
Sham+KMV
28 kD
75 kD
43 kD
VEGF
Ang-1
β-Actin
Relative levels
2.0
1.5
1.0
0.5
0.0
**
**
VEGF
Ang-1
Sham
Sham+KMV
B
Retinas
Sham
Sham+KMV
28 kD
75 kD
43 kD
VEGF
Ang-1
β-Actin
Relative levels
1.5
1.0
0.5
0.0
**
**
VEGF
Ang-1
Sham
Sham+KMV
C
Kidneys
Sham
Sham+KMV
28kD
75kD
43kD
VEGF
Ang-1
β-Actin
Relative levels
1.5
1.0
0.5
0.0
**
**
VEGF
Ang-1
Sham
Sham+KMV
D
Lungs
Sham
Sham+KMV
28kD
75kD
43kD
VEGF
Ang-1
β-Actin
Relative levels
1.5
1.0
0.5
0.0
**
**
VEGF
Ang-1
Sham
Sham+KMV

Figure S12

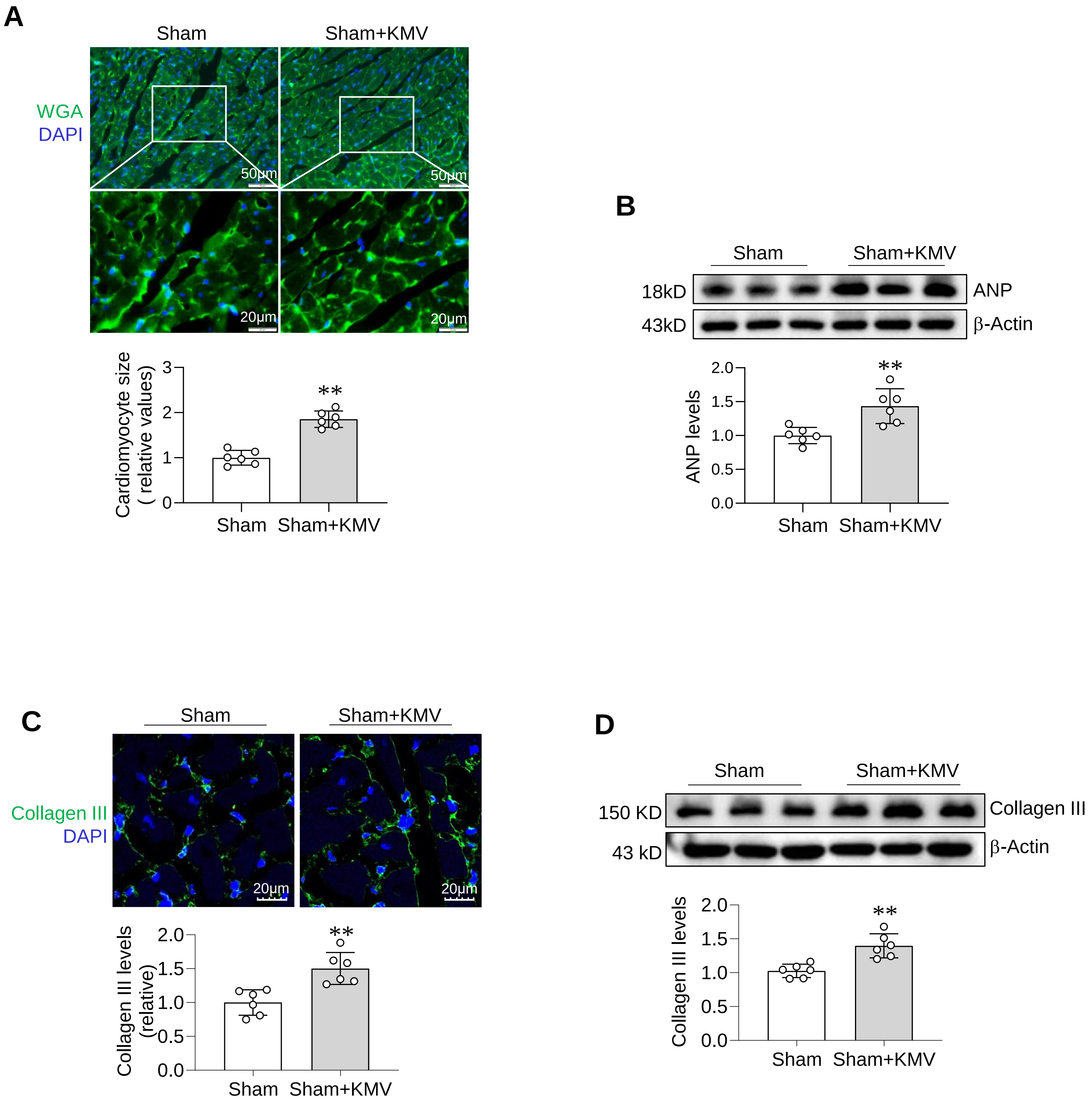
A
Sham
Sham+KMV
WGA
DAPI
50μm
50μm
20μm
20μm
Cardiomyocyte size
(relative values)
3
2
1
0
**
Sham
Sham+KMV
B
Sham
Sham+KMV
18kD
ANP
43kD
β-Actin
ANP levels
2.0
1.5
1.0
0.5
0.0
**
Sham
Sham+KMV
C
Sham
Sham+KMV
Collagen III
DAPI
20μm
20μm
Collagen III levels
(relative)
2.0
1.5
1.0
0.5
0.0
**
Sham
Sham+KMV
D
Sham
Sham+KMV
150 KD
Collagen III
43 kD
β-Actin
Collagen III levels
2.0
1.5
1.0
0.5
0.0
**
Sham
Sham+KMV

Figure S13

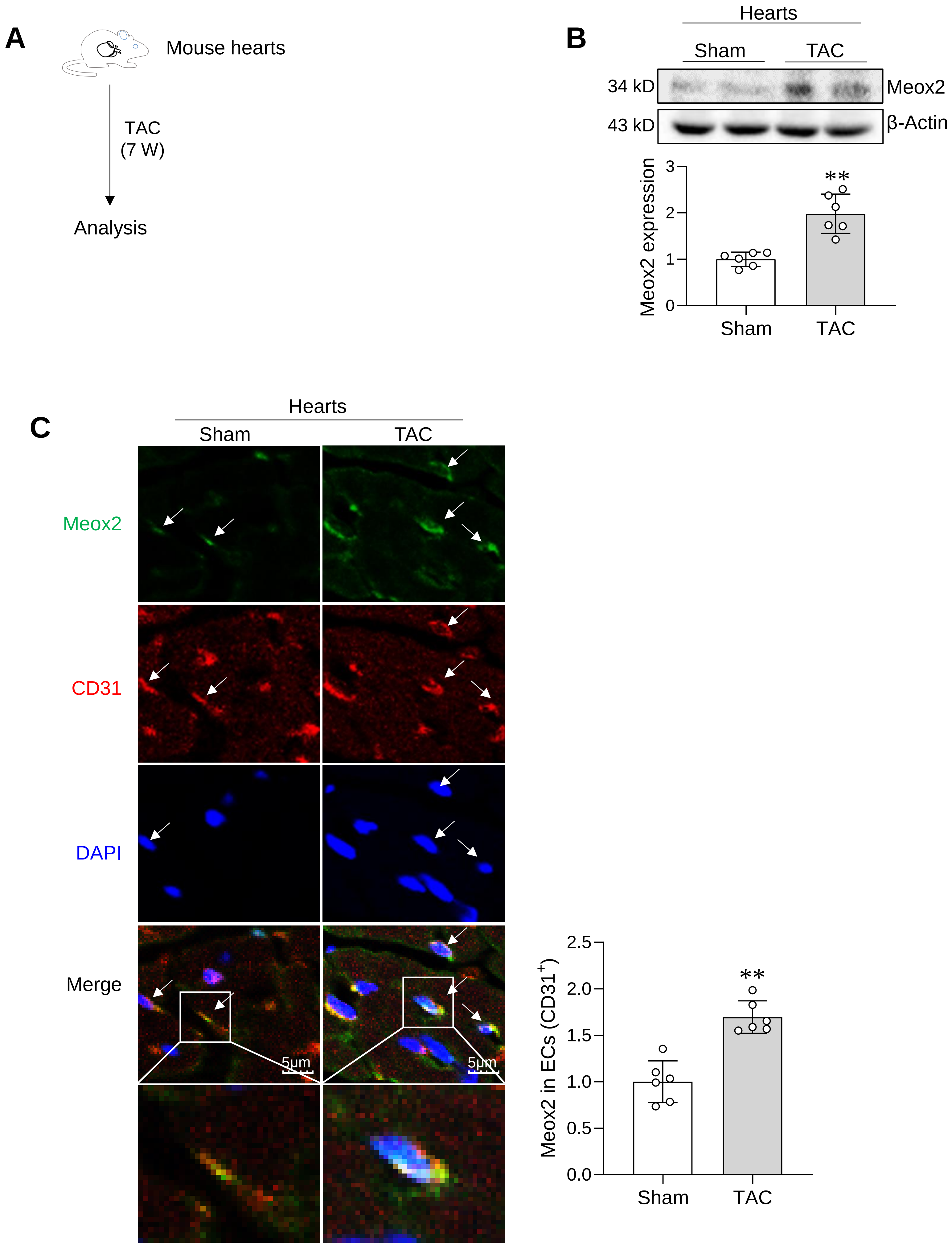

A
Mouse hearts
TAC
(7 W)
Analysis
B
Hearts
Sham
TAC
34 kD
Meox2
43 kD
β-Actin
Meox2 expression
3
2
1
0
**
Sham
TAC
C
Hearts
Sham
TAC
Meox2
CD31
DAPI
Merge
5μm
5μm
Meox2 in ECs (CD31+)
2.5
2.0
1.5
1.0
0.5
0.0
**
Sham
TAC

Figure S14

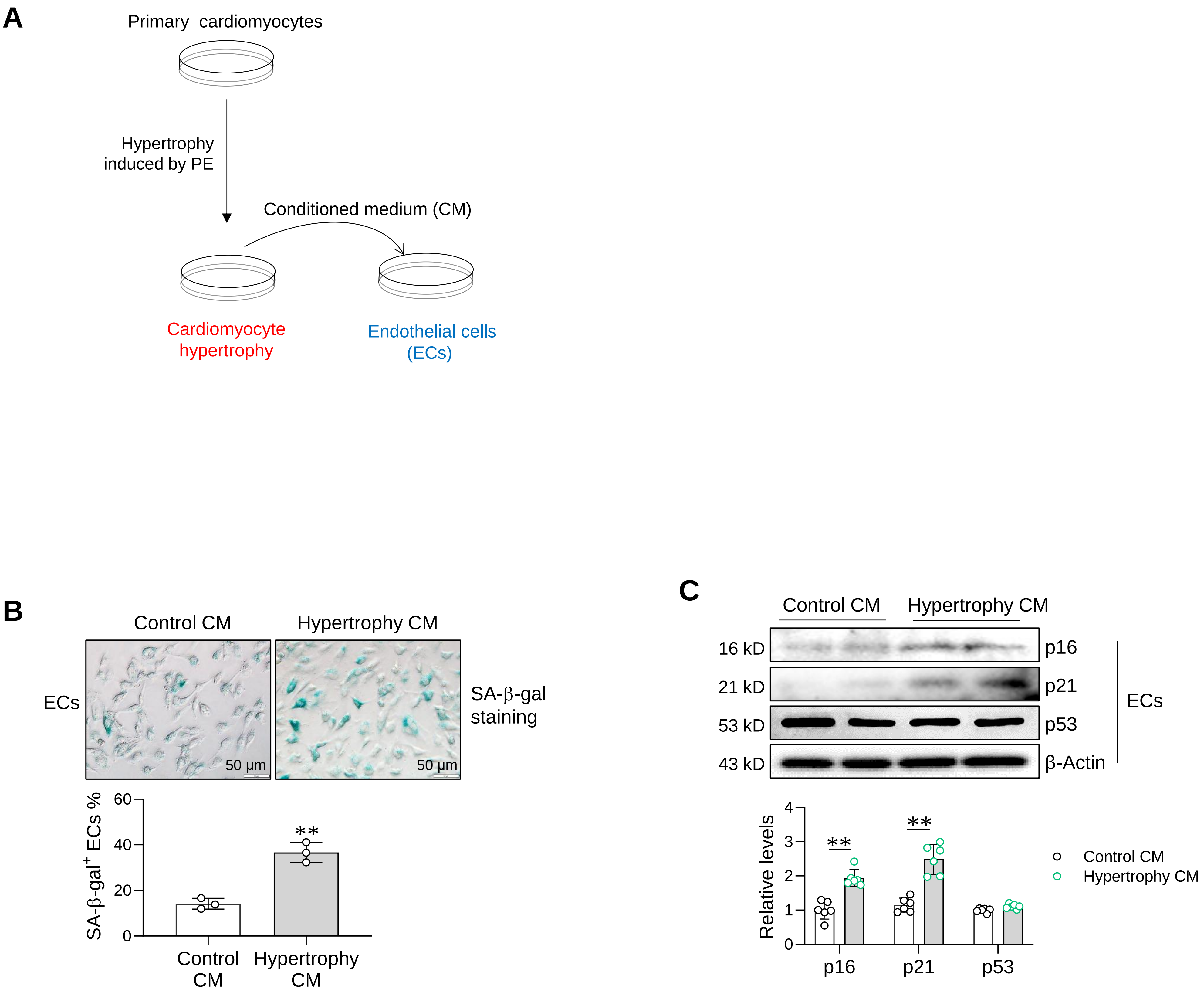
A
Primary cardiomyocytes
Hypertrophy
induced by PE
Conditioned medium (CM)
Cardiomyocyte
hypertrophy
Endothelial cells
(ECs)
B
Control CM
Hypertrophy CM
ECs
SA-β-gal
staining
50 μm
50 μm
SA-β-gal+ ECs %
60
40
20
0
**
Control
CM
Hypertrophy
CM
C
Control CM
Hypertrophy CM
16 kD
21 kD
53 kD
43 kD
p16
p21
p53
β-Actin
ECs
Relative levels
4
3
2
1
0
**
**
p16
p21
p53
Control CM
Hypertrophy CM

Figure S15

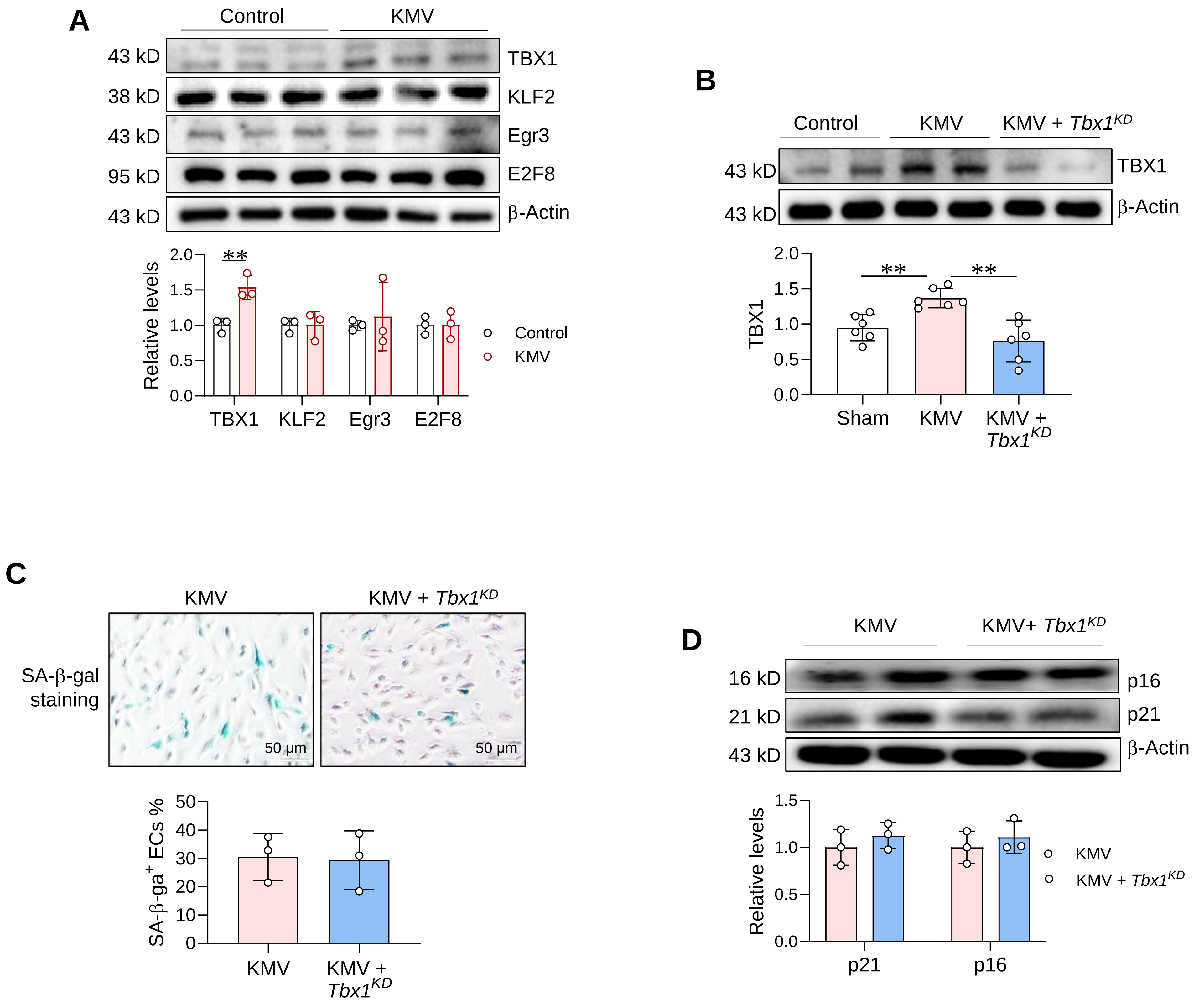
A
Control
KMV
43 kD
38 kD
43 kD
95 kD
43 kD
TBX1
KLF2
Egr3
E2F8
β-Actin
Relative levels
2.0
1.5
1.0
0.5
0.0
**
TBX1
KLF2
Egr3
E2F8
Control
KMV
B
Control
KMV
KMV + Tbx1KD
43 kD
43 kD
TBX1
β-Actin
TBX1
**
**
Sham
KMV
KMV + Tbx1KD
C
KMV
KMV + Tbx1KD
SA-β-gal staining
50 μm
50 μm
SA-β-ga+ ECs %
50
40
30
20
10
0
KMV
KMV + Tbx1KD
D
KMV
KMV+ Tbx1KD
16 kD
21 kD
43 kD
p16
p21
β-Actin
Relative levels
1.5
1.0
0.5
0.0
p21
p16
KMV
KMV + Tbx1KD

Figure S16

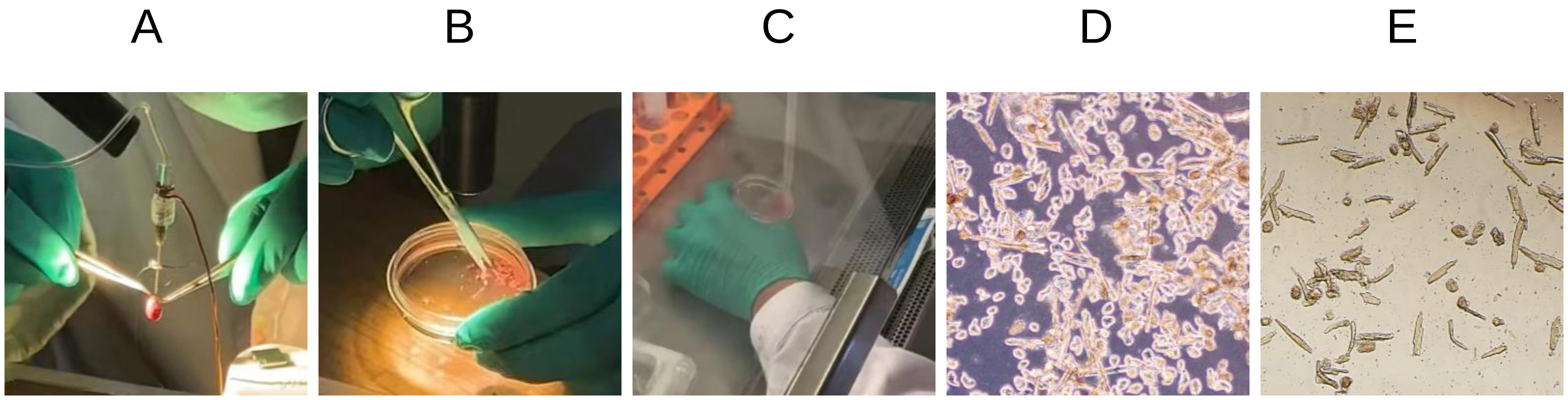

Figure S17

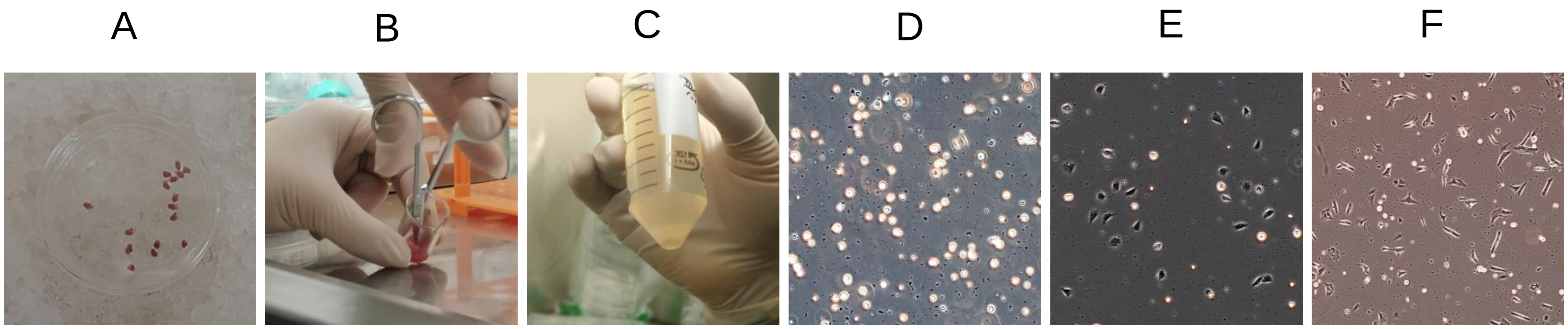

Figure S18

Supplementary tables

Table S1. Echocardiographic measurements

Group	Sham	TAC	TAC+KMV
IVSs, mm	1.25 ± 0.14	1.14 ± 0.05[a]	1.08 ± 0.05[c]
IVSd, mm	0.75 ± 0.07	0.82 ± 0.06	0.80 ± 0.03
LVIDs, mm	2.09 ± 0.10	3.01 ± 0.14[b]	3.69 ± 0.13[c]
LVIDd, mm	3.26 ± 0.09	4.05 ± 0.13[b]	4.61 ± 0.16[c]
LVPWs, mm	1.15 ± 0.12	1.09 ± 0.10[a]	0.95 ± 0.06
LVPWd, mm	0.82 ± 0.09	0.76 ± 0.05[b]	0.72 ± 0.03
LV Mass, mg	98.52 ± 14.07	117.42 ± 12.09[b]	136.50 ± 13.84[c]
LV Mass Corrected, mg	78.82 ± 11.25	93.93 ± 9.67[a]	109.20 ± 11.07[c]
HR, bpm	463.17 ± 0.09	466.50 ± 28.74	448.50 ± 20.47

Abbreviations used: IVSs, LV end-systolic septum thickness; IVSd, LV end-diastolic thickness; LVIDs, LV end-diastolic dimension; LVIDd, LV end-systolic dimension; LVPWs, LV end-systolic posterior wall thickness; LVPWd, LV end-diastolic posterior wall thickness; HR, heart rate; KMV, 3-Methyl-2-oxovaleric acid; SEM, Standard Error of mean. Data presented as mean ± SD. [a]$P < 0.05$ compared to sham. [b]$P < 0.01$ compared to sham. [c]$P < 0.01$ compared to TAC, n = 6/groups

Tables S2. Reagents used in extraction of adult cardiomyocytes

Perfusion buffer		
**Reagents**	**Final concentration**	**Dosage**
NaCl	130 mM	0.456 g
KCl	5 mM	22.4 mg
NaH_2PO_4	0.5 mM	3.6 mg
HEPES	10 mM	0.13 mg
Glucose	10 mM	0.108 mg
BDM	10 mM	60.7 mg
Taurine	10 mM	75.1 mg
$MgCl_2$	1 mM	5.71 mg
ddH_2O		60 ml
Total		60 ml
Adjust PH to 7.8, filtering required		

Tables S3. Reagents used in extraction of adult cardiomyocytes

Collagenase buffer and calcium-contained collagenase buffer		
**Collagenase buffer**		
**Reagents**	**Final concentration**	**Dosage**
perfusion buffer		50 ml
Type 2 collagenase	2.4 mg/ml	120 mg
**Calcium-contained collagenase buffer**		
**Reagents**	**Final concentration**	**Dosage**
collagenase buffer		35 ml
$CaCl_2$	1 mM	15 µl

Tables S4. Reagents used in extraction of adult cardiomyocytes

Plating medium		
**Reagents**	**Final concentration**	**Dosage**
FBS	10%	1 ml
BDM	1 mg/ml	10 mg
Penicillin/streptomycin	1×	100 μl
MEM		8.9 ml
**Total**		10 ml

Tables S5. Reagents used in extraction of adult cardiomyocytes

Maintenance medium		
**Reagents**	**Final concentration**	**Dosage**
BSA	0.5%	50 mg
ITS-A	1×	100 μl
CD lipid concentrate	1×	100 μl
Penicilin/streptomycin	1×	100 μl
MEM		9.7 ml
**Total**		10 ml

Table S6. SiRNA sequence used in the experiments

Species	Gene name		Sequence (5'-3')
Human	*Meox2*	Sense	GCAUUUACCAAAGAGCAAATT
		Antisense	UUUGCUCUUUGGUAAAUGCTT
	Tbx1	Sense	CCUUCCAAGUGAAGCUCUUTT
		Antisense	AAGAGCUUCACUUGGAAGGTT

Table S7. Primers used in the experiments

Species	Gene name		Sequence (5'-3')
Human	*Tbx1*	Forward	ACGACAACGGCCACATTATTC
		Reverse	CCTCGGCATATTTCTCGCTATCT
	Klf2	Forward	CTACACCAAGAGTTCGCATCTG
		Reverse	CCGTGTGCTTTCGGTAGTG
	E2f8	Forward	ATCTGCCTTGACGAAGTGGC
		Reverse	GGCGTACTTATTCTCCTCCCC
	Egr3	Forward	GACATCGGTCTGACCAACGAG
		Reverse	GGCGAACTTTCCCAAGTAGGT
	Meox2	Forward	GGCAAGAGGAAAAGCGACAG
		Reverse	ATCTCGTATCGCCTCAGTCTG
	Sox18	Forward	CCCAACTACAAGTACCGGCC
		Reverse	GCTGCAGTTGAGGTACTGGT
	Pparg	Forward	TACTGTCGGTTTCAGAAATGCC
		Reverse	GTCAGCGGACTCTGGATTCAG
	Prox1	Forward	AAAGGACGGTAGGGACAGCAT
		Reverse	CCTTGGGGATTCATGGCACTAA
	Elk3	Forward	GAGAGTGCAATCACGCTGTG
		Reverse	GTTCGAGGTCCAGCAGATCAA
	Hes1	Forward	CCTGTCATCCCCGTCTACAC
		Reverse	CCTGTCATCCCCGTCTACAC

Table S8. Antibodies used in the experiments

Antibodies	Source	Company	Catalog No.
anti-Angiopoietin-1	Rabbit	Abcam	ab8451
anti-CD31	Mouse	BD Biosciences	550274
anti-E2F8	Rabbit	BYabscience	BYab-6685
anti-EGR3	Mouse	SantaCruz	sc-390967
anti-KLF2	Rabbit	ProteinTech	23384-1-AP
anti-Meox2 for ChIP	Rabbit	SantaCruz	sc-376748
anti-Meox2	Rabbit	Immunoways	YT2821
anti-p16	Rabbit	Bioworld Technology	BS1265
anti-IgG	Rabbit	ABclonal	AC005
anti-p21	Rabbit	ProteinTech	10355-1-AP
anti-p53	Rabbit	Bioworld Technology	BS3156
anti-TBX1	Rabbit	BYabscience	BYab-02076
anti-VEGF	Rabbit	Millipore	07-1420
anti-VEGFR2	Rabbit	ProteinTech	26415-1-AP
anti-VE-cadherin	Mouse	ProteinTech	66804-1-Ig
anti-p-VE-cadherin	Rabbit	Abways	CY6552
anti-α-Actinin	Mouse	SantaCruz	sc-17829
anti-β-Actin	Mouse	ProteinTech	66009-1-Ig

Table S9. Primers used in ChIP-PCR

Species	Gene name		Sequence (5'-3')
Human	*p21*	Forward	GTGACTCATCCTAAGTGGGCA
		Reverse	AGCATTGTCTTGACTGAAACG
	p16	Forward	CCTATCCCCTAGTGATGTCAT
		Reverse	TGTATGGTATGGGCTATTGCT
	Vegf-a	Forward	GCCTTAGGACACCATACCGAT
		Reverse	GGAACAAAGTTGGGGCTCTGA
